# An invasive zone in human liver cancer identified by Stereo-seq promotes hepatocyte–tumor cell crosstalk, local immunosuppression and tumor progression

**DOI:** 10.1038/s41422-023-00831-1

**Published:** 2023-06-19

**Authors:** Liang Wu, Jiayan Yan, Yinqi Bai, Feiyu Chen, Xuanxuan Zou, Jiangshan Xu, Ao Huang, Liangzhen Hou, Yu Zhong, Zehua Jing, Qichao Yu, Xiaorui Zhou, Zhifeng Jiang, Chunqing Wang, Mengnan Cheng, Yuan Ji, Yingyong Hou, Rongkui Luo, Qinqin Li, Liang Wu, Jianwen Cheng, Pengxiang Wang, Dezhen Guo, Waidong Huang, Junjie Lei, Shang Liu, Yizhen Yan, Yiling Chen, Sha Liao, Yuxiang Li, Haixiang Sun, Na Yao, Xiangyu Zhang, Shiyu Zhang, Xi Chen, Yang Yu, Yao Li, Fengming Liu, Zheng Wang, Shaolai Zhou, Huanming Yang, Shuang Yang, Xun Xu, Longqi Liu, Qiang Gao, Zhaoyou Tang, Xiangdong Wang, Jian Wang, Jia Fan, Shiping Liu, Xinrong Yang, Ao Chen, Jian Zhou

**Affiliations:** 1 Zhongshan-BGI Precision Medical Center, Zhongshan Hospital, Fudan University, Shanghai, China; 2 BGI-Southwest, BGI-Shenzhen, Chongqing, China; 3 BGI-Shenzhen, Beishan Industrial Zone, Shenzhen, Guangdong China; 4 Department of Liver Surgery & Transplantation, Liver Cancer Institute, Zhongshan Hospital, Fudan University; Key Laboratory of Carcinogenesis and Cancer Invasion, Ministry of Education, Shanghai, China; 5 BGI-Hangzhou, Hangzhou, Zhejiang China; 6 College of Life Sciences, University of Chinese Academy of Sciences, Beijing, China; 7 Department of Pathology, Zhongshan Hospital, Fudan University, Shanghai, China; 8 Department of Pathology, Ruijin Hospital, Shanghai Jiao Tong University School of Medicine, Shanghai, China; 9 National Facility for Protein Science in Shanghai, Zhangjiang Lab, Shanghai Advanced Research Institute, Chinese Academy of Sciences, Shanghai, China; 10 Guangdong Provincial Key Laboratory of Genome Read and Write, Shenzhen, Guangdong China; 11 Department of Pulmonary and Critical Care Medicine, Zhongshan Hospital, Fudan University, Shanghai, China; 12 James D. Watson Institute of Genome Science, Hangzhou, Zhejiang China; 13 Shenzhen Key Laboratory of Single-Cell Omics, BGI-Shenzhen, Shenzhen, Guangdong China; 14 JFL-BGI STOmics Center, Jinfeng Laboratory, Chongqing, China; 15 State Key Laboratory of Genetic Engineering, Fudan University, Shanghai, China

**Keywords:** Cancer microenvironment, Tumour heterogeneity

## Abstract

Dissecting and understanding the cancer ecosystem, especially that around the tumor margins, which have strong implications for tumor cell infiltration and invasion, are essential for exploring the mechanisms of tumor metastasis and developing effective new treatments. Using a novel tumor border scanning and digitization model enabled by nanoscale resolution-SpaTial Enhanced REsolution Omics-sequencing (Stereo-seq), we identified a 500 µm-wide zone centered around the tumor border in patients with liver cancer, referred to as “the invasive zone”. We detected strong immunosuppression, metabolic reprogramming, and severely damaged hepatocytes in this zone. We also identified a subpopulation of damaged hepatocytes with increased expression of serum amyloid A1 and A2 (referred to collectively as SAAs) located close to the border on the paratumor side. Overexpression of CXCL6 in adjacent malignant cells could induce activation of the JAK-STAT3 pathway in nearby hepatocytes, which subsequently caused SAAs’ overexpression in these hepatocytes. Furthermore, overexpression and secretion of SAAs by hepatocytes in the invasive zone could lead to the recruitment of macrophages and M2 polarization, further promoting local immunosuppression, potentially resulting in tumor progression. Clinical association analysis in additional five independent cohorts of patients with primary and secondary liver cancer (*n* = 423) showed that patients with overexpression of SAAs in the invasive zone had a worse prognosis. Further in vivo experiments using mouse liver tumor models in situ confirmed that the knockdown of genes encoding SAAs in hepatocytes decreased macrophage accumulation around the tumor border and delayed tumor growth. The identification and characterization of a novel invasive zone in human cancer patients not only add an important layer of understanding regarding the mechanisms of tumor invasion and metastasis, but may also pave the way for developing novel therapeutic strategies for advanced liver cancer and other solid tumors.

## Introduction

Solid tumors are complex, highly heterogeneous ecosystems in which cancer cells interact with various cell types including immune cells and stromal cells, as well as the extracellular matrix (ECM).^1–3^ Determining how solid tumor heterogeneity is established and the functional consequences is essential.^1,4,5^ The tumor margin areas, the areas in which tumor cells invade paratumor tissues and come into direct contact with other cells, are the most active regions for tumor cell infiltration and invasion.^1,4,6^ Thus, comprehensive knowledge about the cell compositions of tumor tissues, their spatial heterogeneities, and their interplay with the tumor microenvironments (TMEs), including the tumor margin areas, will not only reveal how tumors develop and metastasize but also accelerate the development of novel cancer therapeutics.^2,4,7–10^

Single-cell RNA sequencing (scRNA-seq) is a powerful tool that can be used to dissect intra- and inter-cellular and molecular dynamics at the single-cell level. It has been widely used to characterize several types of solid tumors and the associated TMEs.^11,12^ However, scRNA-seq alone cannot provide spatial information.^13^ Further, due to the lack of multi-region sampling, intratumoral spatial heterogeneities at the single-cell resolution remain poorly understood.^14–16^ Recently, we developed SpaTial Enhanced REsolution Omics-sequencing (Stereo-seq), providing nanoscale resolution (diameter, 220 nm/spot), expandable detection areas (10 mm × 10 mm), and the capacity to capture a few hundred spots of data per cell by combining DNA nanoball (DNB) patterned array chips and RNA in situ hybridization.^17–20^ Stereo-seq enables the in-depth characterization of functional and positional information for entire tumor ecosystems at the single-cell level, and precisely uncovers the cell composition, distribution, and cell–cell communications in the TME, especially in the tumor margin areas.

Liver cancers, including hepatocellular carcinoma (HCC) and intrahepatic cholangiocarcinoma (ICC), are some of the most common and aggressive tumor types, with high global incidence and mortality rates due to limited and ineffective treatment options.^21–23^ The liver is also a common site for distant metastasis from other cancers, particularly lung, pancreatic, and colorectal cancers.^21–23^ In this study, using Stereo-seq and scRNA-seq, we obtained a comprehensive landscape view of the tumor ecosystems and cell–cell interactions in liver cancer after analyzing multiple regional sites, including the tumor tissues (T), tumor margin areas (M), paratumor tissues (P), and normal or metastatic lymph nodes (LN). After integrating Stereo-seq data with scRNA-seq, constructing a tumor border scanning and digitization model, and conducting additional bioinformatic analyses, we detected a high degree of cellular and transcriptional heterogeneities in a 500 µm-wide invasive zone, which is defined as the region within 250 µm on both sides of the tumor border. Within the zone, intense suppression of the local immune microenvironment, metabolic reprogramming of tumor cells, and severely damaged hepatocytes with high expression of serum amyloid A1 and A2 (SAA1, SAA2, referred to collectively as SAAs) were noted. Mechanistically, the enhanced expression of SAAs in hepatocytes (Hep1 cells) was induced by activation of the JAK-STAT3 pathway, which was triggered by the secretion of C-X-C Motif Chemokine Ligand 6 (CXCL6) by invasive tumor cells. Furthermore, Hep1 cells localized in the invasive zone could secrete SAAs to regulate Formyl Peptide Receptor 1^+^ (FPR1^+^) macrophage recruitment and induce M2 polarization through SAAs-TLR2 axis, resulting in local immunosuppression and tumor progression. We confirmed our findings in five additional cohorts of patients with primary and metastatic liver cancer. Additionally, in vivo experiments employing both primary HCC and colon adenocarcinoma liver metastasis mouse models demonstrated that, adenovirus-associated virus (AAV)-mediated knockdown of *Saas* in hepatocytes significantly reduced macrophage accumulation around the tumor border and delayed tumor growth. Using high-resolution and spatially-resolved transcriptomics, we precisely characterized the distinctive local ecosystem of a 500 µm-wide invasive zone in liver cancer, which provided meaningful biological insights clarifying the mechanisms of tumor invasion and may support the development of novel therapeutic strategies for solid tumors.

## Results

### Spatially-resolved transcriptomics of four tissue regions in human primary liver cancer

**Fig. 1:**
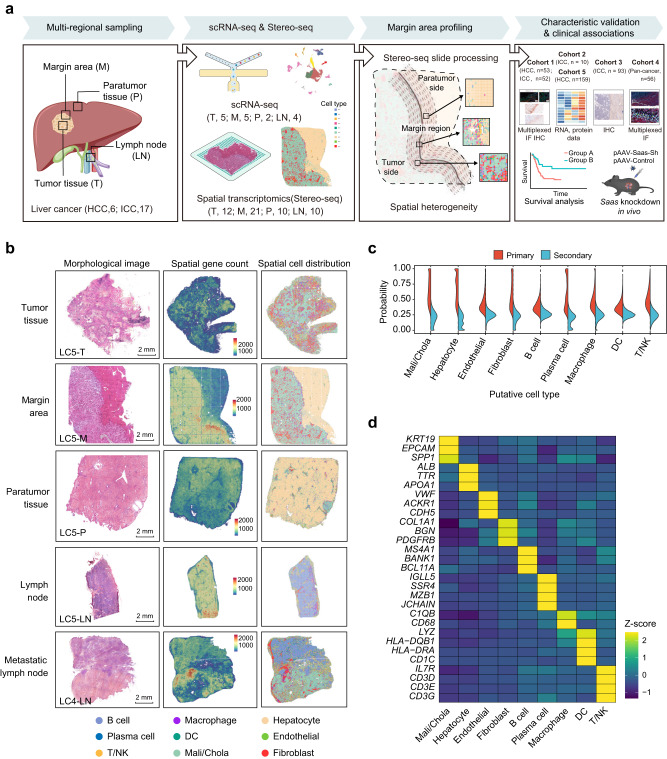
Spatially-resolved transcriptomic profiles in multiple regional sites in primary human liver cancer. **a** A summary of the study. The spatial transcriptomics (Stereo-seq, 53 samples) and scRNA-seq (16 samples) acquisition workflow for 23 patients with liver cancer (HCC, *n* = 6; ICC, *n* = 17) were analyzed as the discovery cohort. Validation cohort 1 included 105 patients with primary liver cancer (HCC, *n* = 53; ICC, *n* = 52). The pan-cancer cohort patients in validation cohort 4 included those with HCC (*n* = 7), ICC (*n* = 20), and those with liver metastasis of colorectal cancer (*n* = 5), pancreatic cancer (*n* = 4), lung cancer (*n* = 5), gallbladder carcinoma (*n* = 5), gastric cancer (*n* = 5), and ovarian cancer (*n* = 5). FFPE tissue blocks from validation cohort 1 were subjected to both multiplexed immunofluorescence (IF) staining and immunohistochemistry (IHC) staining. Specimens from validation cohorts 2, 3, and 4 were subjected to RNA-seq, IHC, and multiplexed IF staining, respectively. The RNA-seq and protein data from validation cohort 5 were from our previously published paper. **b** H&E staining, gene count maps, and cell type maps of different sites (T, M, P, and LN) in samples from patients with liver cancer (LC4 and LC5). **c** The probabilistic inference of cell types at captured spatial transcriptomic spots (50 × 50 bins/spot, 25 µm × 25 µm squares). **d** Heatmap showing the expression levels of marker genes for different cell types in annotated spatial spots. T, tumor tissue; M, margin area; P, paratumor tissue; LN, lymph node; Mali/Chola, malignant cells or cholangiocytes.

To systematically characterize the complex transcriptional architecture of human liver cancer, we processed fresh or freshly frozen T and P, M, and normal or metastatic LN using Stereo-seq with high resolution (diameter of 220 nm/spot) and expandable areas (10 mm × 10 mm), and also analyzed some samples by scRNA-seq (discovery cohort, Fig. 1a; Materials and methods). We generated Stereo-seq datasets for 98 slides from 53 samples (T, 12; M, 21; P, 10; LN, 10) obtained from 21 patients who had been pathologically diagnosed with primary liver cancer (HCC, *n* = 6; ICC, *n* = 15). 16 samples (T, 5; M, 5; P, 2; LN, 4) from 5 patients (HCC, *n* = 1; ICC, *n* = 4) were used for scRNA-seq analysis. There were samples from 3 patients that were analyzed by both Stereo-seq and scRNA-seq (LC11, HCC; LC12 and LC13, ICC). Detailed clinicopathological and data generation information are provided in Supplementary information, Tables S1, S2.

The scRNA-seq data from 33,111 cells were obtained and integrated with a recently published ICC scRNA-seq dataset^24^ to establish an unbiased reference expression fingerprint of cell types to define the spatial tomographies of different cell populations in Stereo-seq slides (Fig. 1a; Supplementary information, Fig. S1a). We used Seurat^25^ to cluster 62,155 qualified cells into 11 main cell types, including T cells (*CD3D*), natural killer cells (NKs) (*KLRF1*), B cells (*MS4A1*), plasma cells (*MZB1*), macrophages (*CD163/CD14*), dendritic cells (DCs) (*CD1C*), cholangiocytes or malignant cells (*KRT19/EPCAM*), hepatocytes (*ALB*), endothelial cells (*CDH5/ENG*), and fibroblasts (*ACTA2*) (Supplementary information, Fig. S1a, b). For Stereo-seq, 2–90 (median = 8) transcripts were detected for each DNB or bin (diameter, 220 nm), with ~2500 bins (50 bins × 50 bins) for each hepatocyte (with a diameter of 25–30 µm) and 900 bins (30 bins × 30 bins) for each malignant cell (with a diameter of 15 µm) (Supplementary information, Fig. S1c). The raw spatial expression matrix was converted into 25 µm × 25 µm pseudo-spots (50 bins × 50 bins/spot, bin50) representing approximately one cell, which resulted in the detection of an average of 589–4642 mRNA molecules and 366–1897 genes per spot (Supplementary information, Table S4). The cell components in each spot (bin50) in the slides were determined by SPOTlight^26^ using scRNA-seq data as the reference, which resulted in the spatial annotation of 9 main cell types (malignant cells and cholangiocytes were assigned as one main cell type; T cells and NK cells were also combined as one main cell type) (Fig. 1b; Materials and methods). We assigned each spot to a specific cell type with the highest probabilistic proportion (Fig. 1c). The higher expression of classical cell type marker genes in defined cell clusters supported the rationale for the cell type annotations used for the spots in these slides (Fig. 1d). Here, the adjacent tissue sections were stained using Hematoxylin and Eosin (H&E) to help confirm the locations of malignant cells and their spatial distribution in Stereo-seq slides.

### Spatial transcriptional heterogeneities in four different regions of primary liver cancer

**Fig. 2:**
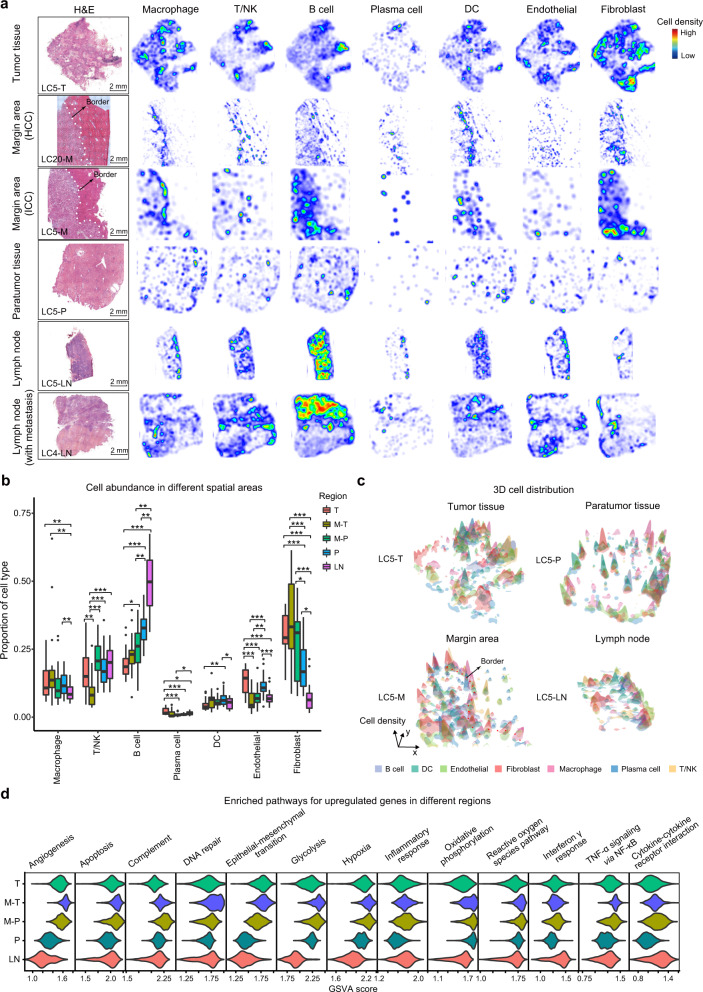
The spatial heterogeneities in tissues from human liver cancer patients. **a** H&E staining and heatmaps of the spatial distribution of the main cell types in multiple regions from patients with liver cancer (LC5-T, M, P, LN; LC20-M; LC4-LN). **b** Box plots showing the percentages of major cell types in all cell components, excluding parenchymal cells, in the four regional sites (T, *n* = 12; M, *n* = 21; P, *n* = 10; LN, *n* = 10) in the Stereo-seq data from 21 patients with liver cancer. Margin areas were further divided into the tumor part (M-T) and paratumor part (M-P) of the margin areas according to the location of the tumor border. **c** Three-dimensional diagrams showing the spatial distribution and aggregation of the main cell types in the four regions (LC5-T, M, P, LN). **d** Violin plots showing the Gene Set Variation Analysis (GSVA) scores of pathways in the five regions (T, *n* = 12; M-T, *n* = 21; M-P, *n* = 21; P, *n* = 10; LN, *n* = 10) based on Stereo-seq data. Student’s *t*-test was used to assess the significance of differences in panel **b**. *P < 0.05; ***P < 0.001. M-T, tumor side of the margin; M-P, paratumor side of the margin.

The cell compositions and spatial distributions were highly heterogeneous in the four regions (T, M, P, and LN), with T/NK cells, B cells and fibroblasts being the most abundant cells aside from the predominant malignant cells and hepatocytes (Fig. 2a). Margin areas were spatially divided into the tumor-adjacent part of margin areas (M-T) and the paratumor part of margin areas (M-P) according to the tumor border identified by pathologists based on adjacent H&E staining images. More endothelial cells and plasma cells were observed in the tumor than in other areas, whereas fibroblasts and macrophages tended to be enriched in the M-T rather than the tumor tissues, and B cells were most abundant in the LNs (Fig. 2b). More fibroblasts were accumulated in the paratumor part of margin areas than in the paratumor tissues (Fig. 2b). Spatially, immune cells such as macrophages were observed to accumulate primarily around the tumor border in margin areas, indicating a distinct immune microenvironment around the border (Fig. 2a, c).

To investigate the microenvironment in different regions, we performed a regional segmentation (1000 bins × 1000 bins or 500 µm × 500 µm per spot, bin1000) to obtain local bulk RNA profiles as one microenvironment unit, with ~400 units present on each slide, and 39,200 units were obtained in total. The enrichment of tight junction, leukocyte transendothelial migration, and ECM receptor interaction pathways was observed in tumor tissues compared with the tumor-adjacent part of margin areas (Supplementary information, Fig. S2a). Pathways related to the epithelial-mesenchymal transition (EMT), the TMEs (e.g., hypoxia, angiogenesis, TGF-β signaling), inflammation and apoptosis (e.g., apoptosis, complement, inflammatory response, reactive oxygen species), glycolysis and oxidative phosphorylation, and immune response (e.g., TNF-α signaling via NF-κB) were highly enriched in the tumor-adjacent part of margin areas compared to tumor tissues (Fig. 2d; Supplementary information, Fig. S2a). Higher enrichment of pathways involved in PI3K/AKT/mTOR signaling, IL6/JAK/STAT3 signaling, and lipid metabolism, including adipogenesis and cholesterol homeostasis, was observed in the paratumor part of margin areas compared to paratumor tissues, while higher enrichment of pathways related to fatty acid metabolism was observed in paratumor tissues compared to the paratumor part of margin areas (Fig. 2d; Supplementary information, Fig. S2a). For LNs, higher enrichment of genes related to the interferon-γ response and cytokine–cytokine receptor interactions were observed compared with other areas (Fig. 2d; Supplementary information, Fig. S2a).

According to the differential expression analysis, the expression levels of genes related to the inflammatory response, including *SAA1*, *SAA2*, *C-reactive protein* (*CRP*), et al., were upregulated in margin areas compared with other areas (Supplementary information, Fig. S2b). These data imply that the tumor margin areas might be a complex region characterized by a hypoxic microenvironment, robust inflammatory responses, and high immune escape.

### The invasive zone is characterized by an immunosuppressive microenvironment, metabolic reprogramming of tumor cells, and severely damaged hepatocytes

**Fig. 3:**
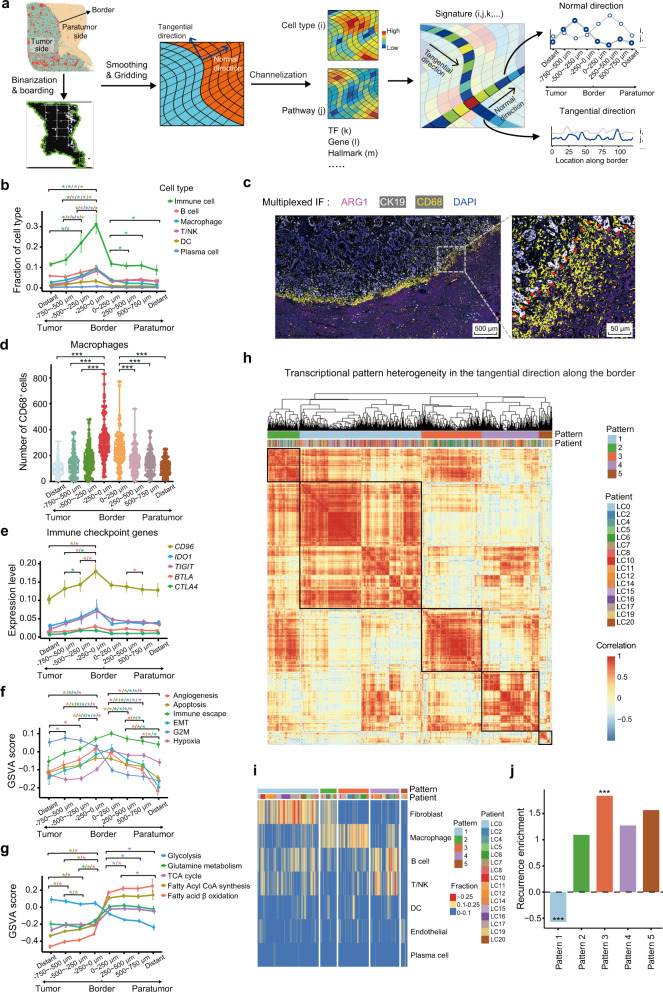
Characteristics along the tangential and normal directions of the tumor border. **a** Schematic diagram of the construction of the tumor border SDM in margin areas using Stereo-seq data. **b** Line graphs showing the average fraction of immune cells and the different subsets (B cells, macrophages, T/NK cells, DC, and plasma cells) among all cell components in different layers around the border of human liver tumors as determined using Stereo-seq data. “Distant” was defined as a 250 µm-wide zone in tumor tissues or paratumor tissues at least 2 mm from the border. **c** Multiplexed IF staining (ARG1, CK19, CD68, and DAPI) of tissue from one representative ICC patient. ARG1, CK19, and CD68 are markers for hepatocytes, malignant cells, and macrophages, respectively. **d** Box plots analyzing the average number of macrophages (CD68^+^ cells) in different layers (1000 µm in axial length) from the paratumor and tumor sides of the border in tissues from 105 liver cancer patients (HCC, *n* = 53; ICC, *n* = 52) from validation cohort 1. **e** Line graphs showing the expression levels of immune checkpoint genes (*CD96, IDO1, TIGIT, BTLA*, and *CTLA4*) in different layers from the border of slides made using tissue specimens from 16 patients with liver cancer based on Stereo-seq data. **f**, **g** Line graphs showing the GSVA scores for different hallmarks of cancer, including angiogenesis, apoptosis, immune escape, EMT, G2M, and hypoxia (**f**) and metabolic pathways, including glycolysis, glutamine metabolism, the tricarboxylic acid (TCA) cycle, fatty acyl CoA synthesis, and fatty acid β-oxidation (**g**) in different layers from the borders of the 16 liver cancer specimens based on Stereo-seq data. **h** Hierarchical clustering showing the transcriptional heterogeneity along the border and illustrating features of cell composition patterns acquired from a total of 2912 equally-divided subregions from the invasive zone in tissues from 16 patients with liver cancer. The features of subregions were grouped into 5 patterns. **i** Heatmap showing the cell type compositions and fractions of the 5 grouped patterns corresponding to panel **h**. **j** Bar charts for the recurrence enrichment score show the prognostic association for each pattern. Student’s *t*-test was used to assess the statistical significance of the differences in panels **b**, **d**–**g** while the Chi-squared test was used in panel **j**. *P < 0.05; **P < 0.01; ***P < 0.001.

Distinct transcriptional landscapes were observed in margin areas, including higher enrichment scores in pathways related to inflammation and the immune response (Fig. 2d; Supplementary information, Fig. S2a, b). Evaluation of Stereo-seq data also revealed the accumulation of macrophages and NK/T cells in margin areas close to the tumor border (Supplementary information, Fig. S3a). To comprehensively dissect the local microenvironment in margin areas, we utilized a novel segmentation method namely scanning and digitization model (SDM) to further investigate the characteristics on both sides of the tumor border (Fig. 3a). Based on Stereo-seq data, we precisely segmented the margin areas along the border into several layers, with each layer representing a 250 µm-wide zone (the width of about ten cells) from the border. Each layer was simultaneously divided into 100 equal parts along the normal direction of the border (Fig. 3a; Materials and methods). Then, we analyzed the fractions and features of the cell components in the six layers on both the paratumor and tumor sides of the border, as well as in the more distant areas represented by the regions 2.00–2.25 mm away from the border (Fig. 3a; Materials and methods). The margin area slides of 16 patients met the acceptability criteria with all the layers covered in the slide and were included for this extensive analysis.

We observed significant enrichment of immune cells and fibroblasts on the tumor side of the border compared with the paratumor side (Fig. 3b; Supplementary information, Fig. S3b). Immune cells were especially enriched within the first layer (0–250 µm) closest to the border from the tumor side, where the immune cell fraction comprised more than 30% of all cell components (Fig. 3b). We also found increased diversity of immune cell abundances along the tangential direction of the border, with the immune cell fractions ranging from nearly 0% to around 100% in different locations along the border (Supplementary information, Fig. S3c). Among the immune cells detected, macrophages, DCs, T/NK cells, and B cells were all more abundant in the area closest to the border on the tumor side compared with the paratumor side (Fig. 3b, c). Moreover, we found that the fractions of macrophages, DCs, T/NK cells, and B cells in all cell components gradually increased from the third layer (−750 µm to −500 µm) to the second layer (−500 µm to −250 µm) and the first layer (−250 µm to 0 µm) on the tumor side of the border. Among them, macrophages were the most enriched in the first layer, with a more than two-fold change (from 3.9% to 8.6%) in the fraction from the third layer to the first layer (Fig. 3b). The results of multiplexed IF staining of 105 primary liver cancer margin areas from the validation cohort 1 (HCC, *n* = 53; ICC, *n* = 52) also revealed that macrophages (CD68^+^ cells) were significantly enriched in the first layer (0–250 µm) on the tumor side (Fig. 3c, d). Specifically, there were significantly increased percentages of anti-inflammatory macrophages (M2-like phenotype) observed in the first layer, accounting for more than two-thirds of the macrophages detected in that layer (Supplementary information, Fig. S3d). We also observed exhausted T cells scattered in this area, with a five-fold increase (from 0.09% to 0.54%) in the fraction of all cells from the third layer to the first layer, indicating an increase in T cell exhaustion as the distance from the border decreased (Supplementary information, Fig. S3e). The expression levels of immune checkpoint genes, including *CTLA4*, *CD96*, and *TIGIT*, were also enriched in the first layer (0–250 µm) on the tumor side, indicating an elevated immunosuppressive status of immune cells in this area (Fig. 3e; Materials and methods). The tumor cells in the first layer also exhibited enhanced immune escape signatures compared with the signatures observed in the outer layer (> 250 µm) (Fig. 3f; Materials and methods). Thus, our results showed distinct local spatial TME features with an immunosuppressive microenvironment around the tumor border, with a marked enrichment of immune cells, including macrophages (particularly M2-like phenotype), T/NK cells, DC cells, and B cells in the first layer (0–250 µm) from the border on the tumor side.

We further characterized the presence of cancer hallmarks and metabolic changes in tumor cells and other cellular components (mainly hepatocytes) in the margin areas. Generally, the tumor cells exhibited a lower hypoxic response, higher glycolysis levels, and higher proliferative capacity (G2M scores) compared with cells on the paratumor side (Fig. 3f, g). Tumor cells in the first layer from the border exhibited increased activation of hypoxic response pathways, angiogenesis, and EMT signatures as compared with those in the layers further from the border (Fig. 3f). The IHC staining of HIF-1α and CD31 that represent hypoxia and vascular cells respectively, also support their enrichment in the first layer of tumor side (Supplementary information, Fig. S3f, g). Increased apoptosis and a lower proliferation capacity were also observed in tumor cells in the closest layer to the border compared with the outer two layers on the tumor side (Fig. 3f). Furthermore, pathways related to fatty acid metabolism, including fatty acyl CoA synthesis and fatty acid β-oxidation, were upregulated in tumor cells in the first layer (Fig. 3g). These results indicated that tumor cells close to the border could actively initiate metabolic reprogramming by upregulating lipid metabolism to obtain additional energy sources, which is critical for tumor invasion. On the paratumor side of the border, pathways related to apoptosis, angiogenesis, proliferation capacity, hypoxia, and glycolysis were significantly enriched in cells among the first layer (0–250 µm) compared with the outer layers (Fig. 3f, g), which implied that there was an enrichment of severely damaged hepatocytes in this area.^27^ Taken together, a distinctive invasive zone — a 500 µm-wide zone centered bilaterally on the tumor border — was identified based on our Stereo-seq data. This invasive zone was characterized by a dominant immunosuppressive environment, enhanced energy supply from fatty acid metabolism and an increased EMT capacity in tumor cells, and severely damaged hepatocytes.

To address possible spatial heterogeneity along the border (Supplementary information, Fig. S3b, c), we further analyzed the diversity and status of cells in the tangential direction along the border in the invasive zone. Based on Stereo-seq data, we further divided the invasive zone into 100 equal subregions in each slide, extracted the cellular components from all subregions excluding malignant cells, cholangiocytes and hepatocytes, and investigated the similarities among ~2912 subregions in the invasive zones from 16 patients (Fig. 3h). The subregions were generally clustered into five patterns (Pattern 1: fibroblasts dominant; Pattern 2: fibroblasts and macrophages dominant; Pattern 3: macrophages dominant; Pattern 4: B cells and T/NK cells dominant; Pattern 5: endothelial cells dominant) based on the cell composition among the subregions (Fig. 3h, i). We found that Pattern 3 was significantly enriched in patients with tumor recurrence (*P* < 0.001) compared to the other patterns, implying that macrophages in the invasive zone might be critical for tumor progression (Fig. 3i, j). In contrast, Pattern 1 was significantly enriched in patients without tumor recurrence (*P* < 0.001, Fig. 3j).

### Identification of a damaged hepatocyte subtype with increased SAAs expression in the invasive zone

**Fig. 4:**
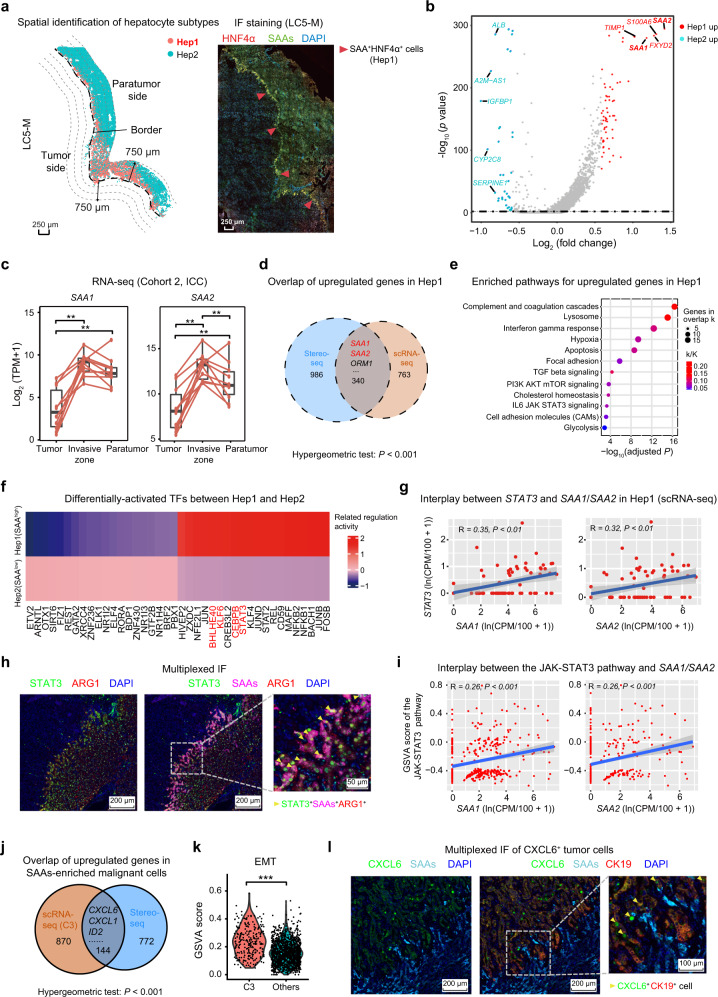
Zone-specific damage of hepatocytes with high expression of SAAs is mediated by JAK-STAT3 activation in the invasive zone. **a** Dot diagram showing the spatial distribution of Hep1 and Hep2 subtypes in the margin areas based on Stereo-seq data (LC5-M) and Hep1 (SAAs^+^ hepatocytes) based on IF staining (HNF4α, SAAs, and 4’-6’-diamidino-2-phenylindole, DAPI) of adjacent frozen slide. HNF4α is a marker to identify hepatocytes. **b** Volcano plot showing differentially expressed genes (DEGs) between two hepatocyte clusters (Hep1 and Hep2) based on Stereo-seq data (LC5-M). The red dots represent genes upregulated in Hep1 and the blue dots represent genes upregulated in Hep2. **c** The expression levels of *SAA1* and *SAA2* in tumors, margin areas (1 cm-wide zone centered on the border) and paratumor tissues from 10 ICC patients from validation cohort 2 determined using bulk-RNA sequencing. **d** The overlap of upregulated genes in Hep1 between the Stereo-seq data (LC2-M) and scRNA-seq data. **e** Bubble diagram showing enriched pathways for commonly upregulated genes in the Hep1 subtype compared with the Hep2 subtype based on the scRNA-seq and Stereo-seq data (LC2-M). k is the number of genes in the intersection of the query set with a set from the database and K is the number of genes in the set from the database. **f** Heatmap of differentially expressed TFs between the Hep1 and Hep2 subtypes based on a SCENIC analysis using scRNA-seq data. **g** Scatter plots showing the interplay between the transcriptional levels of *STAT3* and *SAA1* (left panel) or *SAA2* (right panel) in Hep1 hepatocytes based on scRNA-seq data. **h** Multiplexed IF staining (ARG1, STAT3, SAAs, and DAPI) showing high expression of STAT3 specifically in SAAs^+^ hepatocytes in the invasive zone (in a representative ICC patient from validation cohort 4). **i** Scatter plots showing the correlation between the GSVA score of the JAK-STAT3 pathway and *SAA1* (left panel) or *SAA2* (right panel) in Hep1 hepatocytes based on the scRNA-seq data. **j** The overlap of upregulated genes in cluster 3 (C3) of malignant cells based on the scRNA-seq data and tumor cells in the Hep1-enriched area indicated by the Stereo-seq data (LC2-M). **k** Violin plot representing the GSVA score of the EMT in C3 and other clusters of malignant cells based on the scRNA-seq data. **l** Multiplexed IF staining (CXCL6, SAAs, CK19, and DAPI) showing high expression of CXCL6 in tumor cells close to SAAs^+^ hepatocytes around the border (ICC patient from validation cohort 4). Student’s *t*-test was used for the analysis in panel **c**, a hypergeometric test was used in panels **d**, **j** and the Wilcoxon test was used in panel **k**. *P < 0.05; **P < 0.01; ***P < 0.001.

Our results showed that cells in the paratumor side (mainly hepatocytes) exhibited an increased inflammatory response and apoptosis levels, indicating that they had sustained severe damage. Previous studies have reported that hepatocyte-derived inflammatory responses could contribute to liver metastasis.^28,29^ To further characterize the inflammatory response of hepatocytes and the role of this response in tumor invasion, we reclustered the hepatocytes from the three layers (0–250 µm, 250–500 µm, and 500–750 µm zones) on the paratumor side. We identified two hepatocyte subtypes (Hep1 and Hep2), where Hep1 exhibited higher expression levels of *SAA1* and *SAA2* (Fig. 4a, b). These Hep1 cells were significantly accumulated within the 250 µm-wide zone closest to the border from the paratumor side, and this was confirmed by multiplexed IF staining for the SAAs protein expression levels (Fig. 4a, b; Supplementary information, Fig. S4a, b). The bulk RNA-seq data from validation cohort 2 also revealed higher *SAA1* and *SAA2* expression levels around the border areas (bilateral sampling of 5 mm-wide tissues along the border) than in the corresponding tumor and paratumor tissues (*n* = 10, Fig. 4c). The IHC results from validation cohort 3 (*n* = 93) also revealed higher SAAs abundances in the invasive zone than in the other areas of the tumor or paratumor tissues (Supplementary information, Fig. S4c).

After reclustering all hepatocytes in the scRNA-seq data, cluster 2, which primarily arose from margin areas with the highest expression levels of *SAA1* and *SAA2*, and cluster 0, which primarily arose from paratumor tissues with the lowest expression levels of *SAA1* and *SAA2*, were designated as Hep1 and Hep2, respectively (Supplementary information, Fig. S4d). There were 340 commonly overexpressed genes between Hep1 and Hep2 based on the Stereo-seq data and scRNA-seq data (Fig. 4d). The higher expression of acute-phase protein genes, including *SAA1*, *SAA2*, *Hemopexin* (*HPX*), *Orosomucoid 1* (*ORM1*), *Orosomucoid 2* (*ORM2*), and several mediators of inflammation including *Apolipoprotein A5* (*APOA5*) and *Complement C3* (*C3*) in Hep1 indicated the damaged and inflammatory status of these cells (Supplementary information, Fig. S4e). The commonly upregulated genes were also enriched in complement and coagulation cascades, apoptosis, and cholesterol metabolism, revealing an innate-immune related response and hepatocyte injury in Hep1 (Fig. 4e).

### The damaged SAAs^+^ hepatocyte subtype is induced by JAK-STAT3 activation

To explore the mechanisms accounting for the high expression of SAAs in the Hep1 subtype, a Single-Cell Regulatory Network Inference and Clustering (SCENIC) analysis^30^ was applied and specific transcription factors (TFs), including *FOSB*, *KLF6*, *NFKB1*, *STAT3*, *CEBPB*, *STAT2*, and *ETV6*, were found to be more active in Hep1 compared with Hep2 based on the scRNA-seq data (Fig. 4f). Some TFs, including KLF6, CEBPB, STAT3, and BHLHE40, were annotated as potential TFs for *SAA1* and *SAA2* in ENCODE^31^ or published data.^28,32^ Positive interplay was only observed between *STAT3* and *SAA1* or *SAA2* in the Hep1 subtype based on scRNA-seq data (R = 0.35, *P* < 0.01 for *SAA1*; R = 0.32, *P* < 0.01 for *SAA2*, Fig. 4g), but none of the others showed a significant relationship (Supplementary information, Fig. S5a). We further validated the higher expression level of STAT3 in SAAs^+^ hepatocytes (Hep1) in the invasive zone by multiplexed IF staining (Fig. 4h). The significant positive correlations between the SAAs expression levels and STAT3 were also observed in the RNA-seq and protein data of adjacent tissues from validation cohorts 2 and 5 (Supplementary information, Fig. S5b). There were also positive correlations between the *SAA1* or *SAA2* expression levels and the Gene set variation analysis (GSVA) score of the JAK-STAT3 pathway in Hep1, implying the role of JAK-STAT3 pathway activation in modulating SAAs’ expression (Fig. 4i).

To further explore the mechanism underlying tumor cell-induced hepatocyte injury, we analyzed the differential gene expression between malignant cells close to Hep1-enriched areas and the remaining areas in the slides (Materials and methods). We found that the malignant cells close to Hep1-enriched regions exhibited enhanced EMT, increased metabolic processes (oxidative phosphorylation), and activated immune responses (IFN-γ response) compared with those close to non-Hep1-enriched regions (Supplementary information, Fig. S6a). By reclustering the malignant cells using scRNA-seq data, we identified a subgroup of tumor cells (cluster 3) with similar gene profiles as the tumor cells primarily from margin areas located adjacent to Hep1 in the Stereo-seq data (Supplementary information, Figs. S6b, c). Cluster 3 exhibited enrichment of genes in several pathways including the EMT, fatty acid metabolism, and inflammatory response, compared with the other clusters (Supplementary information, Fig. S6c). In addition, the genes identified by scRNA-seq as upregulated in cluster 3 overlapped with the Stereo-seq data, with 144 genes identified as commonly upregulated, such as *CXCL1*, *ITGAV*, *ID2*, *CRP*, and *CXCL6* et al., which are enriched in EMT, inflammatory response and fatty acid metabolism related pathways (Fig. 4j, k; Supplementary information, Fig. S6c). Among these, CXCL6 was previously reported to activate the JAK-STAT3 signaling pathway.^33,34^ We speculated that it might mediate SAAs expression in Hep1 via the JAK-STAT3 pathway. Multiplexed IF staining showed that CXCL6^+^ tumor cells were located near the SAAs^+^ hepatocytes in the invasive zone, which highly expressed STAT3 and C-X-C Motif Chemokine Receptor 2 (CXCR2), the receptor of CXCL6^35^ (Fig. 4h, l; Supplementary information Fig. S6d). We also found that there was a positive correlation between the expression levels of *STAT3* and *CXCR2* in the bulk RNA-seq data of margin areas (Supplementary information, Fig. S6e). Of note, when primary murine hepatocytes were treated with mouse recombinant GCP-2/CXCL6 protein, there was upregulation of the transcriptional expression of *Saa1*, *Saa2*, and genes involved in the JAK-STAT3 pathway, including *Jak1*, *Jak2*, *Jak3*, and *Stat3*, which occurred in a concentration-dependent manner, as well as pSTAT3, and the inhibition of CXCR2 (SB225002) or STAT3 (Stattic) significantly suppressed the pSTAT3 and decrease the expression level of *Saa1* and *Saa2* (Supplementary information, Fig. S6f, g).

Collectively, our data indicate that liver tumor cells can induce the overexpression of SAAs in hepatocytes through the secretion of CXCL6 and subsequent activation of the JAK-STAT3 pathway in the invasive zone.

### The SAAs^+^ hepatocyte subtype contributes to the recruitment and M2 polarization of macrophages

**Fig. 5:**
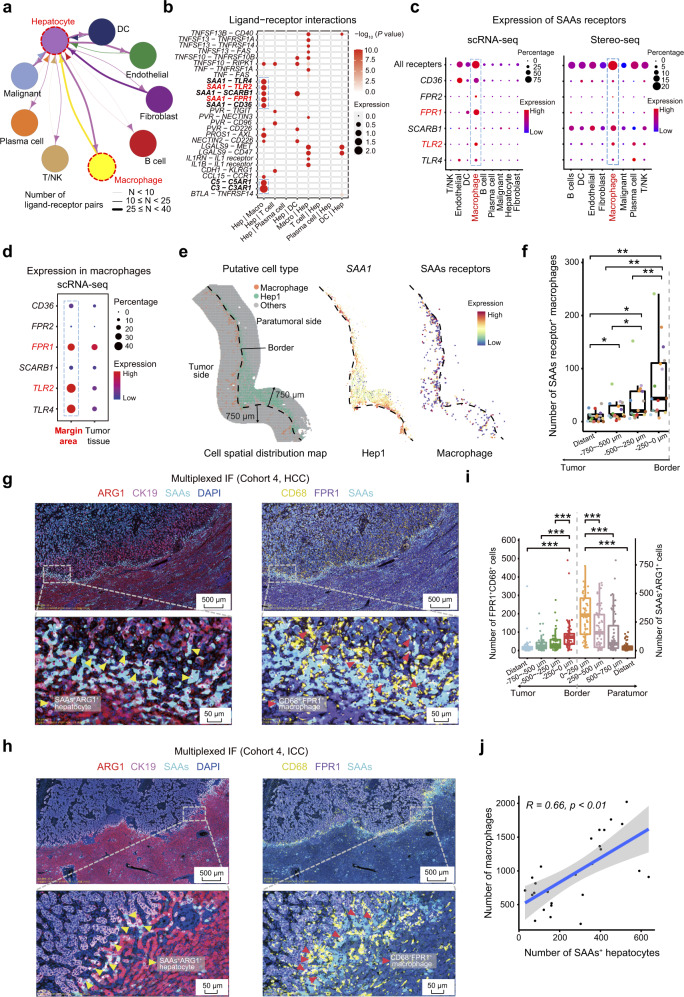
High expression of SAAs was induced in damaged hepatocytes through interactions between CXCR2 and the CXCL6^+^ tumor cells in the invasive zone. **a** The cell–cell interactions between hepatocytes and other cell types indicated by the scRNA-seq data. The line thickness represents the number of significant ligand–receptor pairs, and the arrow points to the cell type that provides the receptor. **b** Bubble plot showing the significant ligand–receptor pairs between hepatocytes and other cell types calculated by CellPhone using scRNA-seq data. **c** Bubble chart showing the gene expression levels of SAAs receptors, including *CD36, FPR1/2, SCARB1, TLR2*, and *TLR4*, in the main cell types based on the scRNA-seq data and Stereo-seq spots (LC2-M). **d** Bubble chart showing the expression levels of SAAs receptor genes in macrophages from the scRNA-seq data of margin areas and tumor tissues from 5 patients. **e** Scatter diagram of the distribution of Hep1 hepatocytes (green dots) and macrophages (red dots) on the LC5-M slide (left panel). Scatter diagram showing the expression of SAAs in Hep1 (middle panel), and the expression of SAAs receptors in macrophages (right panel) on the LC5-M slide. The dash lines represent the border. **f** Box plots analyzing the average number of SAAs receptor (*CD36, FPR1/2, SCARB1, TLR2*, and/or *TLR4*)-positive macrophages in different layers on the tumor side of the border in specimens from 16 liver cancer patients. **g**, **h** Multiplexed IF staining (ARG1, CK19, FPR1, CD68, SAAs, and DAPI) showing co-aggregation of FPR1^+^ macrophages (FPR1^+^CD68^+^ cells) and SAAs^+^ hepatocytes (SAAs^+^ARG1^+^ cells) in the invasive zone of HCC and ICC samples from validation cohort 4. **i** Quantitative analysis of the numbers of FPR1^+^ macrophages (FPR1^+^ CD68^+^ cells) and SAAs^+^ hepatocytes (SAAs^+^ARG1^+^ cells) in different layers (1000 µm in axial length) in the margin areas of 56 liver cancer patients including 27 cases of primary liver cancer and 29 cases of secondary liver cancers (validation cohort 4). “Distant” was defined as a 250 µm-wide zone in tumor tissues or paratumor tissues at least 2 mm from the border. **j** Scatter plot illustrating the correlations between the number of macrophages and the number of SAAs^+^ hepatocytes in the invasive zone (1000 µm in axial length) of samples from 27 primary liver cancer patients from validation cohort 4. The paired Student’s *t*-test was used in panels **f**, **i**. *P < 0.05; **P < 0.01; ***P < 0.001.

Based on spatial cell-bin segmentation^20^ with nucleic acid staining and the identification of cell types and status using margin areas (LC12-M), we further validated the spatial aggregation of SAAs^+^ hepatocytes, SAAs receptor^+^ macrophages, and CXCL6^+^ tumor cells close to the border (Supplementary information, Fig. S6h). To explore the functions of the SAAs^+^ hepatocytes (Hep1) in the invasive zone, we analyzed cell–cell interactions by observing ligand–receptor pairs using the CellPhoneDB based on the scRNA-seq data from margin areas (Materials and methods). The strongest bidirectional communications were observed between hepatocytes and macrophages, with more than 25 significant interacting ligand–receptor pairs (Fig. 5a). Notably, strong cell–cell communications between macrophages and hepatocytes were observed through SAA1–SAAs receptors (FPR1, TLR2, TLR4, SCARB1, and CD36), as well as the C3–C3AR1 and C5–C5AR1 axis (Fig. 5b). The expression of SAAs receptors genes,^36,37^ including *FPR1*, *FPR2*, *TLR4*, *TLR2*, *SCARB1*, and *CD36*, was enriched in macrophages, which was confirmed by the scRNA-seq and Stereo-seq data (Fig. 5c). Both the expression levels and the fractions of cells expressing these genes encoding SAAs receptors were higher in macrophages from margin areas than those from the paired tumor area (Fig. 5d). In addition, macrophages that expressed genes encoding SAAs receptors were spatially accumulated in close proximity to cells of the Hep1 subtype in the invasive zone, and further quantitative analysis revealed the accumulation of SAAs receptor^+^ macrophages in the first layer (0–250 µm from the border) on the tumor side, supporting that there is recruitment of macrophages via the secretion of SAAs by damaged hepatocytes in the invasive zone (Fig. 5e, f).

The location of FPR1^+^ macrophages close to SAAs^+^ hepatocytes in the invasive zone was observed in multiplexed IF staining of validation cohort 4, which included specimens of both primary liver cancer (HCC, 7; ICC, 20) and liver metastasis from different cancers (colorectal cancer, 5; pancreatic cancer, 4; lung cancer, 5; gallbladder carcinoma, 5; gastric cancer, 5; ovarian cancer, 5) (Fig. 5g, h; Supplementary information, Fig. S7a–f). Notably, quantitative analysis revealed significantly more FPR1^+^ macrophages and SAAs^+^ hepatocytes in the invasive zone as compared with other areas in primary liver cancer as well as in the aforementioned metastatic liver cancer (Fig. 5i). A strong correlation between the number of macrophages and SAAs^+^ hepatocytes was also observed in the invasive zone of primary liver cancer (R = 0.66, *P* < 0.01, Fig. 5j). Cell migration assays using THP-1 cells and CD14^+^ peripheral blood mononuclear cells (PBMCs) also demonstrated the role of SAAs in facilitating macrophages migration (Supplementary information, Fig. S8a, b). Besides, hepatocyte supernatant of murine primary hepatocytes in the low chamber of migration assay could significantly enhance the migration of the mouse macrophages namely RAW264.7 cells compared to medium control (Supplementary information, Fig. S8c). Furthermore, the administration of an FPR1 inhibitor namely HCH6-1 could significantly inhibit macrophages migration induced by SAAs in RAW264.7 cells (Supplementary information, Fig. S8d).

**Fig. 6:**
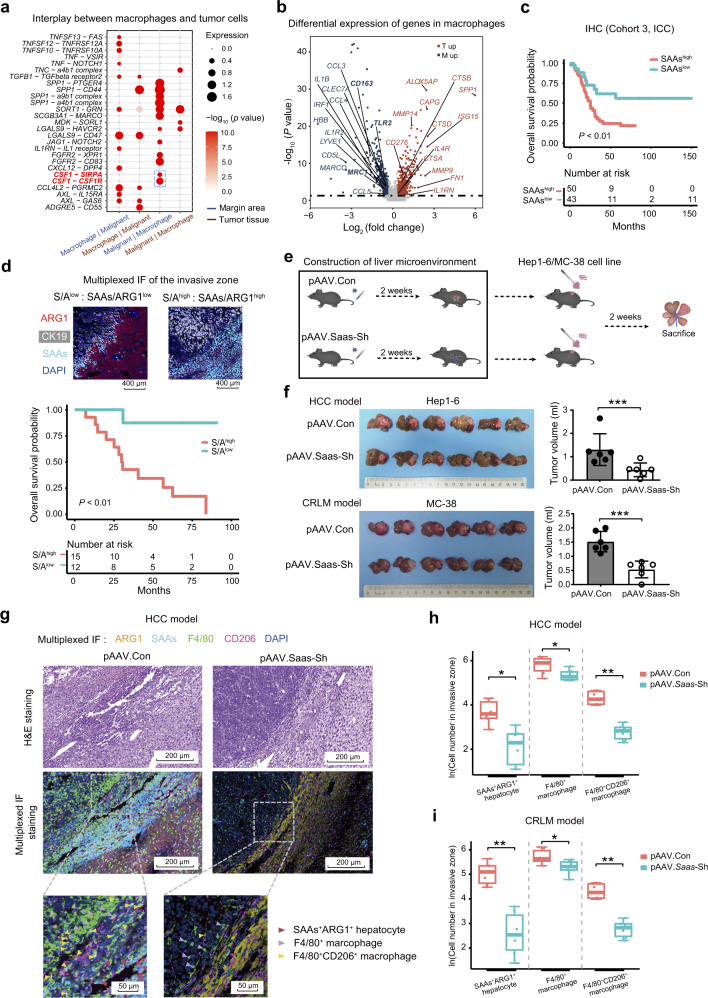
High expression of SAAs by damaged hepatocytes in the invasive zone promoted liver tumor progression. **a** Bubble plot revealing enriched ligand–receptor pairs between macrophages and tumor cells in margin areas identified using scRNA-seq data. **b** Volcano plot showing the genes that were differentially expressed in macrophages based on the scRNA-seq data from 5 patients. The red dots represent genes upregulated in macrophages from tumor sites and the blue dots represent genes upregulated in macrophages from margin areas. **c** OS curves of 93 patients with ICC from validation cohort 3 grouped by SAAs expression in the invasive zone determined using IHC staining. **d** Representative images of the low and high SAAs/ARG1 ratio patients, and OS curves of 27 patients with primary liver cancer from validation cohort 4 grouped by the SAAs/ARG1 ratio determined using multiplexed IF staining (ARG1, CK19, SAAs, and DAPI). **e** Schematic diagram of the construction of the mouse models by implanting tumor tissue sections acquired from subcutaneous tumors (generated using Hep1-6 and MC-38 cell lines) after AAV tail vein injection of pAAV9-Con group (control group) and pAAV9-*Saas*-Sh group (experiment group). **f** Images of the livers with implanted tumors and qualification of the tumor volume in the AAV-Con and AAV-*Saas*-Sh groups generated using the Hep1-6 and MC-38 cell lines. **g**–**i** Representative H&E staining, multiplexed IF staining (ARG1, SAAs, F4/80, CD206, and DAPI) images (**g**) and quantitative cell number of mouse HCC model (**h**) and mouse CRLM (colorectal liver metastases) model (**i**) showing the accumulation of M2 macrophages (F4/80^+^CD206^+^ cells) and SAAs^+^ hepatocytes (SAAs^+^ARG1^+^ cells) in the invasive zone (1000 µm in axial length) in mice from the pAAV9-Con group or pAAV9-*Saas*-Sh group. F4/80 and CD206 are markers of macrophage and M2-type in mice. The Log-rank test was used to assess the data in panels **c** and **d**. Student’s *t*-test was used in panel **f**. The paired Student’s *t*-test was used in panels **h**, **i**. *P < 0.05; **P < 0.01; ***P < 0.001.

An analysis of the ligand–receptor interaction using scRNA-seq data further suggested that the hepatocytes and macrophages in the invasive zone could also interact via SAAs-TLR2 interactions (Fig. 5b), which have been shown to polarize macrophages to an M2-like phenotype.^38^ Our data also showed that malignant cells could directly polarize macrophages toward the M2-like phenotype through CSF1–CSF1R or CSF1–SIRPA interactions in the invasive zone (Fig. 6a). The macrophages in the invasive zone exhibited an M2-like phenotype with increased expression of *CD163, MRC1*, and SAAs receptor *TLR2*, whereas macrophages in the tumor tissue exhibited features of ECM remodeling and increased expression of *MMP9* and *MMP14* (Fig. 6b). In addition, macrophages in the invasive zone also exhibited upregulated expression of multiple cytokines and chemokine genes, such as *CCL3*, *CCL4*, and *IL1B* (Fig. 6b). The multiplexed IF staining results from 105 primary liver cancer patients (validation cohort 1) and IHC results from an additional 93 ICC patients (validation cohort 3) also revealed that M2 phenotype of macrophages accumulated in the invasive zone compared to the other areas of the tumor tissues or paratumor tissues (Supplementary information, Fig. S9a, b). Further studies showed that the addition of SAAs to cultured CD14^+^ PBMCs could upregulate the transcriptional expression of M2 macrophage marker genes, including *MRC1*, *IL1-Rn*, and *IL-10* (Supplementary information, Fig. S9c). The increased fraction of M2 phenotype (CD68^+^CD206^+^) macrophages induced by SAAs were also detected by flow cytometry and TLR2 inhibitor treatment could significantly decrease the percentage of M2 phenotype (Supplementary information, Fig. S9d). In summary, injured hepatocytes induced by malignant cell could recruit M2 polarizing macrophage in the invasive zone by secreting SAAs.

### The SAAs^+^ hepatocyte subtype is associated with tumor progression

We next explored the clinical significance of the damaged hepatocytes and the recruited macrophages in the invasive zone. The increased expression levels of *SAA1* and *SAA2* in margin areas were significantly associated with a worse overall survival (OS) in ICC patients from validation cohort 2 (*P* < 0.05 for *SAA1* and *P* < 0.05 for *SAA2*; Supplementary information, Fig. S10a, b). The prognostic significance of SAAs in the invasive zone was also confirmed in 93 other ICC patients (validation cohort 3) by IHC analysis (*P* < 0.01 for OS; Fig. 6c). Specifically, a high ratio of SAAs^+^ hepatocytes in the invasive zone was significantly correlated with a worse OS in patients with primary (*P* < 0.01) and secondary liver cancer (*P* < 0.05) (validation cohort 4; Fig. 6d; Supplementary information, Fig. S10c). Moreover, we found a strong positive correlation between SAAs expression in the invasive zone and the paratumor tissues by IHC staining of the samples from 93 patients with ICC from validation cohort 3, indicating that the damaged hepatocytes in the paratumor tissues reflected the damage detected in the invasive zone (*R* = 0.75, *P* < 0.001; Supplementary information, Fig. S10d). The increased SAAs expression levels in the paratumor tissues in validation cohort 3 were also significantly correlated with a shorter OS (*P* < 0.05 for OS; Supplementary information, Fig. S10e), and this relationship was further confirmed at the transcriptional and protein levels in validation cohort 5 (Supplementary information, Fig. S10f, g).

Using pAAV-*Saas*-Sh and pAAV-Con C57BL/6 N mouse models with orthotopically implanted liver tumors derived from subcutaneous tumors (HCC cell line, Hep1-6; colon adenocarcinoma cell line, MC-38), we demonstrated that the specifically knockdown of *Saa1* and *Saa2* expression in hepatocytes by pAAV-*Saas*-Sh significantly inhibited primary and secondary liver tumor growth in vivo (1.31 ± 0.68 mL vs 0.44 ± 0.29 mL, *P* < 0.05 for Hep1-6 cell line; 1.52 ± 0.33 mL vs 0.53 ± 0.27 mL, *P* < 0.05 for MC-38 cell line; Fig. 6e, f; Supplementary information, Fig. S10h, i). Further multiplexed IF staining also confirmed that SAAs^+^ hepatocytes, macrophages (F4/80^+^ cells), and M2 macrophages (F4/80^+^ CD206^+^ cells) were enriched around the border of liver tumors (invasive zone) from pAAV-Con primary and secondary mouse model (Supplementary information, Fig. S10j, k). Moreover, *Saas* knockdown in hepatocytes significantly abrogated SAAs expression in hepatocytes and reduced their subsequent recruitment of macrophages and M2 polarization in the invasive zone (Fig. 6g–i; Supplementary information, Fig. S10j).

**Fig. 7:**
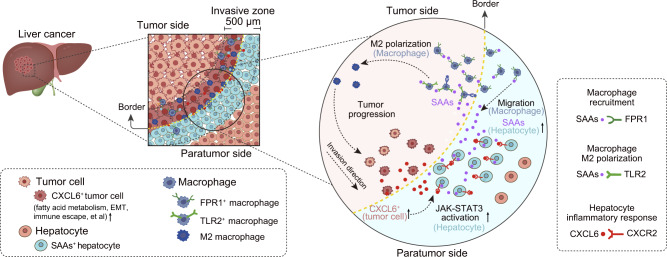
Schematic diagram of the invasion zone. Schematic diagram showing the local ecosystem where tumor cells with high invasiveness (CXCL6^+^ tumor cells), damaged hepatocytes (SAAs^+^ hepatocytes) and recruited FPR1^+^ macrophages polarizing into the M2 phenotype interacted in the 500 µm-wide invasive zone, contributing to tumor progression.

Collectively, our data suggest that macrophages are specifically recruited and polarized to the M2 phenotype via the secretion of SAAs by damaged hepatocytes in the invasive zone (Fig. 7). This leads to increased immunosuppression of the local microenvironment and promotes tumor progression. The knockdown of *Saas* inhibited tumor growth, suggesting that SAAs inhibition may represent a potential therapeutic strategy for primary and secondary liver cancer.

## Discussion

Dissecting and understanding the cancer ecosystem are essential for exploring the mechanisms of tumor metastasis and developing effective new treatments in liver cancer. Previous studies of primary liver cancer including HCC- and ICC-related studies using scRNA-seq have paved the way for the identification of unidentified cell types, states, and potential functions. Some T cell subtypes (including exhausted CD8^+^ T cells, non-classical CD8^+^CD4^+^ T cells, and relapsed tumor-enriched CD161^+^CD8^+^ T cells), LAMP3^+^ migration DCs, endothelial cells driving immunosuppressive macrophages, CCL4^+^ tumor-associated neutrophils and VEGFA^+^ malignant cells are highlighted and well discussed.^16,39–44^ However, lack of spatial information, the particular interactions in some concerned regions such as the invasive front have not been decoded yet. Besides, hepatocytes are also rarely discussed in scRNA-seq studies due to the reason that they are too fragile to be captured with enough numbers using scRNA-seq. In this study, we integrated spatially-resolved transcriptomics with subcellular resolution and scRNA-seq to determine the cellular composition and transcriptional architecture within four regional sites (T, M, P, and LN) in tumors from patients with liver cancer. The tumor margin area was the most active region, representing a sophisticated and dynamic TMEs^4,28,45^ characterized by the presence of and interactions among highly invasive tumor cells (CXCL6^+^ tumor cells), damaged hepatocytes (SAAs^+^ hepatocytes), and recruited FPR1^+^ macrophages, all of which contributed to tumor progression (Fig. 7). Our findings advance the current understanding of how the local immune environment shapes tumor progression and reveal the existence of active crosstalk among malignant cells, immune cells, and hepatocytes, which could facilitate the development of novel therapeutic strategies for liver cancers.

Using our high-resolution, spatially-resolved technology, we characterized the spatial transcriptomic heterogeneity around the border, a feature that has been largely overlooked due to the limitations of the existing research tools.^9,46–49^ While analyzing the spatial heterogeneity of the margin area, we surprisingly identified a distinctive, 500 µm-wide “invasive zone” centered on the tumor border with three features that distinguished the area from other regions: (1) In this invasive zone, tumor cells exhibited metabolic reprogramming to fatty acid metabolism, accounting for greater aggressiveness, as indicated by increased activation of the hypoxic response pathway, angiogenesis, immune escape, and EMT signatures compared with the other areas. (2) Increased inflammatory responses and damaged hepatocytes were found close to the border, mainly due to the direct invasion of malignant cells. (3) There was a local immunosuppressive microenvironment in the invasive zone, characterized by increased expression of immune checkpoint genes including *CTLA4*, *CD96*, and *TIGIT*, creating a favorable environment for tumor progression. Based on our observation that the most profound variations in the immune microenvironment and metabolic changes in the tumor cells were between the second layer (250–500 µm) and the first layer (0–250 µm) on the tumor side of margin area, and similar trends in the inflammatory responses of hepatocytes were found on the paratumor side, we concluded that the 500 µm-wide zone centered on the tumor border (250 µm on either side) was distinct from other areas. Thus, we redefined the 500 µm-wide zone centered on the tumor borderline as “the invasive zone,” which was more reasonable than the previously defined 1000 µm-wide area centered on the border.^1,50–52^

Furthermore, we found that the invasive zone in liver cancers harbored a distinct local tumor ecosystem with a complex interplay among the cell components (Fig. 7). We characterized an enriched tumor cell cluster (CXCL6^+^ tumor cells) that featured enhanced EMT, altered metabolic processes, and activated immune responses in this invasive zone compared to other areas. Additionally, the CXCL6 secreted by the tumor cell cluster could induce damage to hepatocytes (SAAs^+^ hepatocytes) via activation of the JAK-STAT3 pathway. We found that the secretion of SAAs by damaged hepatocytes facilitated tumor invasion by recruiting FPR1^+^ macrophages and subsequent M2 polarization. This is consistent with a previous study reporting that hepatocytes with upregulated SAA1 and SAA2 expression provided a pro-metastatic niche for liver metastasis in a mouse model of pancreatic cancer.^28^ Alternatively, complement components secreted by hepatocytes, including C3a and C5a, could recruit macrophages via C3AR1/C5AR1,^53^ and CSF1 secreted by tumor cells could promote macrophage M2 polarization via SIRPA/CSF1R^54^ in the invasive zone, indicating that the invasive zone is a sophisticated ecosystem with multi-directional interactions.

The characterization of this invasive zone provides significant clinical insights for prognostic prediction and suggests several potential therapeutic targets for solid tumors. The footprints left by tumor invasion on the paratumor liver tissues, namely SAAs expression by damaged hepatocytes, macrophage infiltration, and metabolic reprogramming of tumor cells in the invasive zone, could serve as useful prognostic indicators of the extent of tumor aggressiveness and risk of recurrence. The findings also suggest that the invasive zone may have potential therapeutic targets, including SAAs and CXCL6, allowing for more precise treatment of liver cancers. Comprehensively defining the local immune ecosystem in the invasive zone would also advance our understanding of the mechanisms underlying tumor metastasis and might facilitate the development of more effective therapeutic strategies for other solid tumors.

Compared with data generated using other spatial transcriptome methods, our Stereo-seq data had unprecedented nanoscale resolution (diameter 220 nm/spot) and expandable detection areas (10 mm × 10 mm), enabling a more precise view of all cell types and cell–cell communications in the target area, as well as expanded spatial transcriptome analyses.^1,9,20,55–57^ Here, we characterized the transcriptional architecture in pseudo-spots using 25 µm × 25 µm squares (50 × 50 bins/spot, bin50), representing approximately one cell. Thus, we could precisely segment each layer of margin areas at the single-cell level and establish a novel segmentation method, SDM, to comprehensively examine the invasive zone (the regions from 0–250 µm on both sides of the tumor border). We also employed SDM to characterize the spatial heterogeneity of the cellular composition and transcriptional architecture in the normal and tangential directions along the tumor border, demonstrating functional spatial heterogeneity. Investigations using this technology in datasets for other solid tumors may identify commonalities among solid tumors or may define tumor type- or location-specific features that can be targeted for cancer prevention or treatment.

Our study did have some limitations. Macroscopic and pathological examinations revealed that a capsule existed around the tumors in 10%–76% of the HCC patients, but the tumor capsule rarely existed in the ICC patients.^58,59^ It is believed that the formation of this fibrous capsule results from tumor–host interactions, and that the capsule acts as a physical barrier, preventing the infiltration of immune cells in the margin area, hindering the interplay among the cells around the border.^58,60^ Thus, we only selected liver cancer patients who lacked a complete tumor capsule upon pathological examination to permit better characterization of the invasive zone. Moreover, although our Stereo-seq technology provided high nanoscale resolution (diameter 220 nm/spot) and the capacity to capture transcripts in a few hundred spots per cell, it was still difficult to identify cell boundaries and to capture exact transcripts at the single-cell level.^20,30,61^ Future technological advances may eliminate these limitations.

In conclusion, we identified and characterized a 500 µm-wide invasive zone centered on the tumor border in liver cancer. This zone was revealed to encompass a sophisticated and dynamic local ecosystem that could determine the risk of tumor invasion and the patient prognosis. Our results also provide evidence that our SDM method is useful for exploring tumor ecosystems, making it possible to characterize the spatial heterogeneity present in cancer, which could inform the development of more precise and effective treatments for liver cancer and other solid tumors.

## Materials and methods

### Study subjects

A total of 23 primary liver cancer patients (HCC, *n* = 6; ICC, *n* = 17) with matched fresh tumor tissues, paratumor tissues, margin area tissues, and lymph node samples were enrolled as the discovery cohort. Formalin-fixed paraffin-embedded (FFPE) tissue blocks of margin area tissues were collected from patients who had undergone liver resection and were pathologically diagnosed with primary liver cancer (HCC, *n* = 53; ICC, *n* = 52) as validation cohort 1. Matched, frozen tumor tissues (at least 2 cm from the tumor border), paratumor tissues (at least 2 cm from the tumor border), and margin area tissues (1 cm-wide zones centered on the tumor border) were collected from 10 ICC patients as validation cohort 2. FFPE tissue blocks of margin area tissues (2 × 2 × 1 cm, 2 cm-wide zones centered on the tumor borders) samples were collected from 93 ICC patients as validation cohort 3. The FFPE tissue blocks of margin area tissues were collected from patients who had undergone liver resection and were pathologically diagnosed with HCC (*n* = 7), ICC (*n* = 20) or secondary liver cancer metastasized from colorectal cancer (*n* = 5), pancreatic cancer (*n* = 4), lung cancer (*n* = 5), gallbladder cancer (*n* = 5), gastric cancer (*n* = 5), or ovarian cancer (*n* = 5) as validation cohort 4 (pan-cancer cohort). Paired frozen tumor tissues and paratumor tissues were collected from 159 HCC patients who had undergone liver resection as validation cohort 5.^62^ The detailed sample and pathological information is shown in Supplementary information, Tables S1, S2, S3. All patients provided informed consent for the collection of clinical information, and the tissue collection protocols were approved by the Institutional Review Board [approval B2018-018(3)] at Zhongshan Hospital, Fudan University.

### scRNA-seq

#### Preparation of single-cell suspensions

Margin area tissues (a 1 cm-wide zone centered on the tumor border) were surgically removed from resected liver lobes from patients with liver cancer and were immersed in 90% Dulbecco’s Modified Eagle medium (DMEM, Gibco, Gaithersburg, MD, USA) with 10% fetal bovine serum (FBS; Gibco), and were transported to the laboratory in a refrigerated container. Suitable small tissue blocks were then cut into pieces, which were transferred to MACS C tubes (Miltenyi Biotec, Bergisch Gladbach, Germany), with 5 mL of digesting enzyme included in a Tumor Dissociation Kit (Miltenyi Biotec). The tissues were then converted into single-cell suspensions using a gentle MACS Dissociator (Miltenyi Biotec). In brief, samples were milled, incubated at 37 °C for 30 min on a shaker, milled again, incubated at 37 °C for 30 min, milled a third time and then filtered through a 70 mm filter in 2% FBS. Finally, the single-cell suspension was centrifuged at 300× *g* for 7 min, and resuspended with cell resuspension buffer at a cell concentration of 1000 viable cells/μL.

#### scRNA-seq library construction

scRNA-seq libraries were prepared using DNBelab C4 system as described previously.^20^ Barcoded mRNA capture beads, droplet generation oil, and the single-cell suspension were loaded into the corresponding reservoirs on the chip for droplet generation. The droplets were gently removed from the collection vial and placed at room temperature for 20 min. Droplets were then broken and collected by the bead filter. The supernatant was removed, and the bead pellet was resuspended with 100 μL RT mix. The mixture was then thermal cycled as follows: 42 °C for 90 min, 10 cycles of 50 °C for 2 min, and 42 °C for 2 min. The bead pellet was then resuspended in 200 μL of exonuclease mix and incubated at 37 °C for 45 min. Afterward, the PCR master mix was added to the bead pellet and thermal cycled as follows: 95 °C for 3 min, 13 cycles of 98 °C for 20 s, 58 °C for 20 s, 72 °C for 3 min, and finally 72 °C for 5 min. Amplified cDNA was purified using 60 μL of AMPure XP beads. The cDNA was subsequently fragmented to 400–600 bp with NEBNext dsDNA Fragmentase (New England Biolabs) according to the manufacturer’s protocol. Indexed sequencing libraries were constructed using the reagents provided in the C4 scRNA-seq kit as follows: (1) post-fragmentation size selection with AMPure XP beads; (2) end repair and A-tailing; (3) adapter ligation; (4) post-ligation purification with AMPure XP beads; (5) sample index PCR and size selection with AMPure XP beads. The barcode sequencing libraries were quantified by Qubit (Invitrogen). The sequencing libraries were sequenced using the DIPSEQ T1 sequencer at the China National GeneBank. The read structure was paired-end with Read 1, covering 30 bases inclusive of the 10 bp cell barcode 1, 10 bp cell barcode 2, and 10 bp unique molecular identifier, and Read 2 containing 100 bases of the transcript sequence, and a 10 bp sample index.

### Methods used for spatial transcriptomic studies

#### Stereo-seq chip preparation

Capture chips were generated following the Stereo-seq protocol.^20^ In brief, to generate the DNB array for in situ RNA capture, we first synthesized random 25-nucleotide coordinate identity (CID) -containing oligonucleotides, circularized with T4 DNA ligase, and splint oligonucleotides. DNBs were then generated by rolling circle amplification and loaded onto the patterned chips (65 mm × 65 mm). Next, to determine the distinct DNB-CID sequences at each spatial location, single-end sequencing was performed on a DNBSEQ-Tx sequencer (MGI Research, Shenzhen, China) with a SE25 sequencing strategy. After sequencing, poly-T and 10 bp molecular identity (MID)-containing oligonucleotides were hybridized and ligated to the DNB on the chip. This procedure produced capture probes containing a 25 bp CID barcode, a 10 bp MID, and a 22 bp poly-T ready for in situ capture. CID sequences, together with their corresponding coordinates for each DNB, were determined using a base calling method according to the manufacturer’s instructions for the DNBSEQ sequencer. After sequencing, the capture chip was split into smaller size chips (10 mm × 10 mm). At this stage, all duplicate CID that corresponded to non-adjacent spots were removed.

#### Tissue sectioning, fixation, staining, and imaging

By avoiding sampling necrotic area and area around great vessels, tumor tissue (1 × 1 cm area, at least 2 cm from the tumor border), margin areas (1 × 1 cm area centered on the tumor border), paratumor tissue (1 × 1 cm area, at least 2 cm from the tumor border), and LN samples were collected and snap-frozen in optical cutting tissue (OCT) compound (Tissue-Tek; Sakura Finetek USA, Torrance, CA, USA). After collection, the tissues were snap-frozen in liquid nitrogen containing prechilled isopentane in Tissue-Tek OCT and transferred to a –80 °C freezer for storage before the experiment. The pre-frozen liver tissues in OCT were transversely sectioned at a thickness of 10 µm using a CM1950 cryostat (Leica, Wetzlar, Germany). Tissue sections adhering to the Stereo-seq chip surface were incubated at 37 °C for 3 min. The tissues were then fixed in methanol and incubated at –20 °C for 40 min. The adjacent tissue sections adhering to the glass slides were stained using H&E. Imaging for both procedures was conducted using a Ti-7 Eclipse microscope (Nikon, Tokyo, Japan).

Tissue patches on the chip were permeabilized using 0.1% pepsin (Sigma-Aldrich, St. Louis, MO, USA) in 0.01 M HCl buffer (pH = 2), incubated at 37 °C for 10 min and then washed with 0.1× SSC buffer (Thermo Fisher Scientific, AM9770) supplemented with 0.05 U/μL RNase inhibitor (New England Biolabs, Ipswich, MA, USA). The RNA released from permeabilized tissues was captured using DNB probes and reverse-transcribed overnight at 42 °C using SuperScript II (10 U/μL reverse transcriptase), 1 mM dNTPs, 1 M betaine solution PCR reagent, 7.5 mM MgCl_2_, 5 mM DTT, 2 U/μL RNase inhibitor, 2.5 μM Stereo-TSO, and 1× First-Strand buffer. After in situ reverse transcription, tissue patches were washed twice with 0.1× SSC buffer and digested with tissue removal solution (STOmics, 1000028505) at 37 °C for 30 min. The cDNA-containing chips were then incubated with 400 μL cDNA release solution (STOmics, 1000028512) for 3 h at 55 °C, and then washed once with 400 μL of 0.1× SSC buffer (Thermo Fisher Scientific, AM9770). All products were purified using 0.8× Ampure XP Beads (Vazyme Biotech, Nanjing, China), and were amplified with KAPA HiFi Hotstart Ready Mix (Roche, Basel, Switzerland) using 0.8 μM cDNA-PCR primers. PCR reactions were conducted as follows: first incubation at 95 °C for 5 min, 15 cycles of 98 °C for 20 s, 58 °C for 20 s, then 72 °C for 3 min, and a final incubation at 72 °C for 5 min. PCR products were purified using 0.6× Ampure XP Beads. The concentrations of cDNA were quantified using a Qubit™ dsDNA Assay Kit (Thermo Fisher Scientific).

#### Library preparation and sequencing

A total of 20 ng cDNA was fragmented with in-house Tn5 transposase at 55 °C for 10 min. The reactions were then terminated by the addition of 0.02% SDS buffer with gentle mixing at 37 °C for 5 min. Fragmentation products were amplified as follows: 25 μL of fragmentation product, 1× KAPA HiFi Hotstart Ready Mix, 0.3 μM Stereo-Library-F primer, and 0.3 μM Stereo-Library-R primer in a total volume of 100 μL with the addition of nuclease-free H_2_O. The reaction was then run as follows: one cycle at 95 °C for 5 min, 13 cycles at 98 °C for 20 s; 58 °C for 20 s and 72 °C for 30 s, and one cycle at 72 °C for 5 min. The PCR products were purified using Ampure XP Beads (Vazyme; 0.6× and 0.2×) for DNB generation and were finally sequenced (a paired-end of 100 bp) using a MGI DNBSEQ-Tx sequencer.

#### Cell clustering

The clustering analysis of the scRNA-seq dataset was performed using Seurat (version 3.2.2) and the R program, and the parameters were manually curated to portray an optimal classification of cell types based on empirical knowledge. Specifically, low quality cells with fewer than 500 detected genes or with more than 6000 genes, as well as those with > 20% mitochondrial counts in data preprocessing were filtered out, and all query genes were guaranteed to be expressed in at least three cells prior to further use. The top 3000 highly variable genes were then selected according to their mean variance ratio for the expression levels after log1p normalization. For downstream clustering and visualization, principal component analysis (PCA)-based dimension reduction was initially performed, and the first 18 principal components (PCs) were extracted for subsequent Louvain clustering to define the cell types (the resolution was set to 0.3). The clustering results were finally characterized in a two-dimensional space using the uniform manifold approximation and projection (UMAP) technique, and the cell types were annotated using known biomarkers that were more highly expressed in a particular cluster (via the FindAllMarkers function with default parameters).

#### Cell type inference of spatial transcriptome spots

To overcome the low RNA capture efficiencies on single DNB spots at a resolution of 500 nm, the raw spatial expression matrix was convoluted into larger pseudo-spots with a 50 × 50 window size (bin50 for short), or more precisely, as 25 µm squares. The cell type composition for each bin50 spot was then inferred by the SPOTlight software^26^ (version 0.1.6) with factorized cell type-specific topic profiles from paired scRNA-seq data. The potential composition of each spot was pruned and renormalized using the top four cell types with respective probabilities in descending order, and the primary cell type was assigned for visualization.

#### Differential gene expression analysis

DEG analysis in each cluster was performed using the FindAllMarkers function of the Seurat package (v3.2.2), and the DEGs between the two groups were detected using the FindMarkers function. The parameter condition was min.pct = 0.1, logfc.threshold = 0.15.

#### Functional enrichment analysis

To identify the biological function(s) of the DEGs in each cluster, we performed gene set enrichment analyses (GSEA) using the Molecular Signatures Database of H (hallmark gene sets, version 7.4) according to a previous publicaton^63^ (GSEA, https://www.gsea-msigdb.org/gsea/index.jsp). To characterize the differences in pathways, as well as biological functions, between tumor cells from SAAs-enriched areas and non-SAAs-enriched areas, the DEGs between the tumor cells in two layers around the border of LC5-M were used. Similarly, the DEGs detected in the two subtypes of hepatocytes in LC5-M were used for the pathway enrichment analysis.

#### Transcription factor analysis

A cell-to-gene signal matrix depicting gene abundances was input into the pySCENIC pipeline^30^ with default settings used to infer statistically active TFs and their targets. First, it inferred co-expression modules using GRNBoost2, a regression per-target approach. Second, it pruned indirect targets from modules using regulatory motif discovery (cisTarget). In brief, enriched motifs were discovered from all genes in co-expression modules. Each remaining TF and its potential direct target were called a regulon. Finally, it used AUCell as a metric to compute the activity of each regulon in each bin. To identify the specific regulons of the two hepatocyte subtypes, we used a Wilcoxon Rank-Sum test to calculate the significance of differences in the TFs between the Hep1 and Hep2 subtype. The specific TFs in Hep1 were displayed by Feature Plots on spots. The related regulation activity scores of differentially activated TFs between Hep1 and Hep2 is the mean scaled values. In principle, each TF activity score of single cells is independently normalized to get the z-score with zero mean and unit variance, then the mean score of Hep1 or Hep2 is calculated.

### Construction of the tumor border SDM

To identify the border of the paratumor and intratumor regions by spatial transcriptome profiling, the tumor section was first processed into a binary image that masked the predicted hepatocyte cells. Only the large area of the liver paratumor tissue was retained as a region of interest, and scatter signals were filtered by window-size pixel thresholding (the threshold was set manually to sweep scatter signals outside regions on different sections), and the boundary pixels were extracted using the Contours function in the Python OpenCV package and initialized into a rough edge. The edge was then smoothed by spline fitting (with 20 degrees of freedom) using the R spline package, and local segmentation and/or rotations were introduced for complex borders that could not be directly fitted (for example, a border graphed as a parabola with a horizontal axis of symmetry that barely fit without a 90° rotation).

After the determination of the border, parallel curved lines were generated by perpendicularly extending lines 250 µm, 500 µm, and 750 µm to both the tumor and paratumor sides, to measure the appropriate width of the invasive zone. Specifically, 6 infiltrating layers (bidirectional) were derived from the border, and each layer was segmented into 100 tiles with approximately equal areas along the border line. The spots/cells were subsequently assigned to the corresponding tiles by calculating the sign of the outer product of their centroid coordinates to each edge of the tiles, and were used to assess the variations of spatial gradients of cellular components and gene expression profiles in both the tangential and normal directions of the border.

### Detection of cell subtypes

#### T cell, B cell, macrophage, and CAF subtypes

To study the subclasses of each cell type, the cell spots identified with higher confidence were extracted from the SPOTlight output.^26^ Counts per million (CPM) normalization was performed for each spot to ensure that the gene relative abundance was comparable between spots, and gene standardization was applied to the empirical biomarkers of corresponding cell subtypes (summarized in Supplementary information, Table S5) to balance the expression levels for those selected marker genes that showed a significantly different expression in corresponding subtypes. The highest average score was then assigned to the spot to represent the most probable subtype for downstream analysis.

### Identification of the SAAs^high^ hepatocyte subtype

The SAAs-abundant hepatocytes (Hep1 cells) were directly distinguished by increasing the resolution of Louvain unsupervised clustering in the integrated scRNA-seq data, and the most significant marker genes (including *SAA1* and *SAA2*), which defined this particular hepatocyte subtype, were intersected into a reference set (GS1). In a similar manner, the countered reference set was generated (GS2), specifying hepatocyte cells without significant expression of SAAs. For the Stereo-seq data, hepatocytes from the invasive zone were unsupervised clustered in LC2-M and LC5-M samples to identify SAAs^high^ or SAAs^low^ hepatocytes; for the invasive zone hepatocytes in other 14 margin area samples, the values of the GS1 and GS2 sets in each bin were calculated by the Addmodulescore function in Seurat, and the SAAs^high^ or SAAs^low^ hepatocytes were defined by their differences in expression.

### Cell type enrichment, gene expression, and tumor hallmark score analysis in margin areas

After the stratification and blocking of margin areas into small tiles with roughly equal areas, the cell type composition, gene expression, and tumor hallmark scores were assessed on these elaborated spatial bulk RNA profiles. In particular, cell types were summarized by the normalized probabilities of each bin50 spot that was inferred by the former SPOTlight results.^26^ The gene expression levels were normalized using the CPM and compared between the tiles and layers on a bulk level. The tumor hallmark scores were generated by extracting the tumor cell spots (using the criteria described for the cell subtype detection) in border-to-intratumor tiles, normalizing them, and comparing them to border-to-peritumor tiles by Gene Set Variation Analysis (GSVA). All results were shown as the percentages of each tile, and statistical analyses were conducted to assess the heterogeneity in both the tangential and normal directions of the leading edge.

### Analysis of cell–cell interactions

To analyze the cellular cross-talk between different cell types in different regions, CellPhoneDB (version 2.0.3),^64^ a public repository of ligand–receptor pairs, was used to identify significant ligand–receptor interactions. The interaction score refers to the mean of the average expression values for all individual ligand–receptor partners in the corresponding interacting pairs for the different cell types. The output of any complex’s expression in CellPhoneDB was calculated by the sum of the expression of the component genes. For different tissues, the cell type-specific ligand–receptor interactions between cell types were identified based on the specific expression of the ligand by one cell type and expression of the corresponding receptor by another cell type.

### Patterns of cell components in the invasive zone

To evaluate the variations of spatial gradients of cellular components along the tangential direction of the border, cell components were extracted after removing the malignant cells and hepatocytes in each tile segmented from the closest layers around the border of samples from 16 liver cancer patients. The cell components of two bilateral tiles around the same location along the border were integrated to calculate the proportion of cell components in each patient, and then the Pearson correlation was determined between the different regions. Finally, the value of the correlation coefficient was used to perform hierarchical clustering on all regions to elucidate the pattern.

### IF staining

FFPE tissue blocks of margin area tissues (2 × 2 × 1 cm, a 2 cm-wide zone centered on the tumor border) were collected from 105 patients who had undergone liver resection and were pathologically diagnosed with primary liver cancer (HCC, *n* = 53; ICC, *n* = 52) for validation cohort 1 and 56 patients who had undergone liver resection and were pathologically diagnosed with HCC (*n* = 7), ICC (*n* = 20), and those with liver metastasis of colorectal cancer (*n* = 5), pancreatic cancer (*n* = 4), lung cancer (*n* = 5), gallbladder carcinoma (*n* = 5), gastric cancer (*n* = 5), and ovarian cancer (*n* = 5) for validation cohort 4. The PANO 7-plex IHC kit (Panovue, Beijing, China) was conducted to multiplexed IF staining of human FFPE tissues according to the manufacturer’s instructions by appling primary antibodies against targets including CD68 (#76437 S, CST, Danvers, MA, USA), CD163 (#ab182422, Abcam, Cambridge, UK), CK19 (#ab52625, Abcam), ARG1 (#93668 S, CST), STAT3 (#ab68153, Abcam), p-STAT3 (phospho Y705) (#ab267373, Abcam), SAA1/2 (#ab207445, Abcam), CXCR2 (#ab225732, Abcam), FPR1 (#ab113531, Abcam), S100P (#ab124743, Abcam), CK20 (#13063 S, Abcam), TTF1 (#ab76013, Abcam), PanCK (#CST4545, CST), and p53 (#CST2527S, CST). The multiplexed IF standard operation procedures of Wisee Bio were applied for mouse FFPE tissues using primary antibodies against targets including F4/80 (#70076 S, CST), ARG1 (#93668 S, CST), SAA1/2 (#ab199030, Abcam), CD206 (#AF2535, R&D Systems, MN, USA). They were followed by incubation with horseradish peroxidase-conjugated secondary antibody and tyramide signal amplification. The slides were then microwave heat-treated after each TSA procedure. Nuclei were stained with DAPI (Sigma-Aldrich) after all the human antigens had been labelled. For each slide, three zones with a width of 500 µm and length of 1000 µm from tumor tissues, paratumor tissues, and the areas centered on the border were selected for image capture and further analysis.

### Bulk RNA extraction and sequencing

For 10 ICC patients who had undergone liver resection and were pathologically diagnosed with ICC in validation cohort 2, matched, frozen tumor tissues (at least 2 cm from the tumor border), paratumor tissues (at least 2 cm from the tumor border), and margin area tissues (1 cm-wide zones centered on the tumor border) were collected. Total RNA from the tumor tissues, margin area tissues, and paratumor tissues were isolated using a RNeasy Mini Kit (Qiagen, Hilden, Germany). Strand-specific libraries were prepared using a TruSeq Stranded Total RNA Sample Preparation kit (Illumina, San Diego, CA, USA) following the manufacturer’s instructions. Briefly, mRNA was enriched with oligo (dT) beads. Following purification, the mRNA was fragmented into small pieces using divalent cations at 94 °C for 8 min. The cleaved RNA fragments were then copied into first strand cDNA using reverse transcriptase and random primers, followed by second strand cDNA synthesis using DNA Polymerase I and RNase H. These cDNA fragments then underwent an end repair process, involving the addition of a single “A” base, followed by ligation of the adapters. The products were then purified and enriched by PCR to create the final cDNA library. Purified libraries were quantified using a Qubit 2.0 fluorometer (Life Technologies, Carlsbad, CA, USA) and confirmed using a 2100 Bioanalyzer (Agilent Technologies, San Jose, CA, USA) to confirm the insert size and calculate the molar concentration.

Clusters were generated by cBot with the library diluted to 10 pM and then sequenced using a NovaSeq 6000 (Illumina). The library construction and sequencing were performed at Shanghai Biotechnology Corporation (Shanghai, China). For each sample, 33–95 million RNA-seq clean reads were obtained using HISAT2 (hierarchical indexing for the spliced alignment of transcripts)^65^ version 2.0.477. Sequencing read counts were calculated using Stringtie (version 1.3.0).^66,67^ The expression levels from different samples were then normalized by the Trimmed Mean of M values method. The normalized expression levels of different samples were converted to fragments per kb of transcript per million mapped (FPKM) fragments.

### Flow cytometric analysis

THP-1 cells namely the human monocytic leukemia cell line (acquired from China National Collection of Authenticated Cell cultures) were differentiated with phorbol 12-myristate 13-acetate (PMA #HY-18739, MedChemExpress, Shanghai, China) with a concentration of 200 ng/mL for two days in RPMI 1640 medium containing 10% FBS. When THP-1 reached confluence, the medium was supplemented with SAAs (#ab50232, Abcam) with a concentration of 500 ng/mL or SAAs of 500 ng/mL combinding with TLR2 inhibitor TLR2-IN-C29 (#S6597, Selleck, Shanghai, China) of 20 µM or CXCR2 inhibitor for 12 h. Collected THP-1 cells were stored in cell staining buffer (#420201, Biolegend, San Diego, CA, USA) and incubated with Zombie NIR™ Fixable Viability Kit (#423106, Biolegend), PE anti-human CD45 antibody (#368510, Biolegend) and PE/Cyanine7 anti-human CD86 antibody (#374210, Biolegend). After fixation and permeabilization of cells using BD Cytofix/Cytoperm™ Fixation/Permeabilization Solution Kit (#B554714, BD Biosciences Pharmingen, San Diego, CA, USA), BV421 anti-human CD68 Y1/82 A (#564943, BD Biosciences Pharmingen) and APC anti-human CD206 (MMR) antibody (#321110, Biolegend) were then applied to stain intracellular markers.

### IHC staining and evaluation

FFPE tissue blocks of margin area tissues (2 × 2 × 1 cm, a 2 cm-wide zone centered on the tumor border) were collected from 93 ICC patients who had undergone liver resection and were pathologically diagnosed with ICC as validation cohort 3. FFPE tissue blocks of margin areas from these 93 patients were used for IHC staining. Primary antibody against targets included SAAs (#ab190802, Abcam) and CD31 (#BX50032, Biolynx, Hangzhou, China), HIF-1α (#ab272693, Abcam). All staining was conducted using the IHC/ISH System (BenchMark GX; Roche, Basel, Switzerland) following the manufacturer’s instructions.

To evaluate the staining index, three zones with a width of 500 µm and a length of 1 mm were selected from tumor areas, areas around the border (the zone centered on the border), and paratumor tissues. The staining index was further acquired using the ImageJ 1.53 (National Institutes of Health, Bethesda, MD, USA) IHC profiler.

### Primary murine hepatocyte isolation and treatment

Primary hepatocytes from male wild-type C57BL/6 mice were isolated using a two-step perfusion method as described previously.^68^ Then, isolated hepatocytes were seeded on collagen-coated culture dishes and used for subsequent experiments. Primary murine hepatocytes were treated with mouse recombinant GCP-2/CXCL6 protein (#ab9925, Abcam; 0 ng/mL, 100 ng/mL, 500 ng/mL, and 1000 ng/mL) for 36 h.

### Isolation and treatment of CD14^+^ PBMCs

Monocytes were purified from the whole blood of healthy blood donors following a protocol approved by the Institutional Review Board at Fudan University, Zhongshan Hospital. Briefly, monocytes were isolated from whole blood by positive sorting using anti-CD14-conjugated magnetic microbeads (Miltenyi Biotec, Bergisch Gladbach, Germany). The monocyte purity was > 90% as assessed by flow cytometry (data not shown). A total of 15 × 10^4^ CD14^+^ PBMCs was added every well of a twelve-well plate, and cells were treated with 200 ng/mL SAAs for 0 h, 6 h, 12 h, or 24 h.

### Transwell migration assay

24-well transwell chambers (8.0 µm pore size; Costar; Kennebunk, ME, USA) were used to determine the migratory abilities of THP-1. 24-well transwell chambers (5.0 µm pore size; Costar) were used to determine the migratory abilities of mouse macrophage cell line RAW264.7. THP-1 cells were pretreated with 200 ng/mL PMA (#HY-18739, MedChemExpress) for two days in RPMI 1640 medium containing 10% FBS. The CD14^+^ PBMCs were pretreated with 200 ng/mL M-CSF (macrophage colony stimulating factor 1; #300-25, PeproTech, Rocky Hill, USA) for four days in RPMI 1640 medium containing 10% FBS. For the migration assays, 5 × 10^4^ induced THP-1 cells or CD14^+^ PBMCs resuspended in FBS-free RPMI 1640 medium were seeded in the upper compartments of transwell chambers. RPMI 1640 medium containing 5% FBS was employed as a chemoattractant in the lower compartments. The THP-1 and induced CD14^+^ PBMCs were treated with different concentrations of SAAs (#ab50232, Abcam): 0 ng/mL, 10 ng/mL, 100 ng/mL, or 500 ng/mL. RAW264.7 cells were treated with different concentrations of SAAs (#2948-SA-025, R&D Systems): 0 ng/mL, 25 ng/mL, 100 ng/mL, or 250 ng/mL. FPR1 inhibitor HCH6-1 (#HY-101283, MedChemExpress, Shanghai, China) was added in the medium with a concentration of 3 μM in the lower compartments of transwell chambers. Hepatocyte supernatant was collected from the 12-well chambers seeded with 80 × 10^4^ murine primary hepatocytes in one chamber with 0.5 mL DMEM medium without foetal bovine serum for 12 h. After 48 h for THP-1 cells and CD14^+^ cells and 24 h for RAW264.7 cells, the cells that passed through the pores in the membrane were fixed with 4% polyformaldehyde and stained with 0.5% crystal violet. After extensive washes with 1× phosphate-buffered saline, images were captured using a light microscope (200× magnification; Olympus Corporation, Tokyo, Japan). Five fields of view were randomly chosen and the average cell number was determined.

### Construction of mouse models with implanted liver tumors, and *Saas* knockdown

Male wild-type C57BL/6 mice (8 weeks old, purchased from Beijing Vital River Laboratory Animal Technology Co., Ltd.) were housed under specific pathogen-free conditions. Animal protocols were reviewed and approved by the Institute of Animal Care and Use Committee of Zhongshan Hospital, Fudan University. All animals received humane care according to the “Guide for the Care and Use of Laboratory Animals” criteria of the National Academy of Sciences (National Institutes of Health publication 86-23, revised 1985). The pAAV9-*Saas*-Sh and control virus were purchased from Shanghai Zorin biotechnology Co., Ltd. A total of 1 × 10^11^ V.G. units of AAV were injected into every mouse via a tail vein (6 mice in the pAAV9-Con group and 6 in the pAAV9-*Saas*-Sh group). Two weeks later, approximately 3 mm × 3 mm × 3 mm tumor tissue sections acquired from subcutaneous tumors (1 × 10^6^ Hep 1-6 cells or MC-38 cells injected subcutaneously 2 weeks earlier in other mice) were implanted into the left lateral lobe of the liver in each mouse. The livers were then harvested 2 weeks later for tumor volume measurement and IF staining of FFPE slides (SAAs, F4/80, and DAPI).

### Mouse SAAs enzyme-linked immunosorbent assay (ELISA)

Murine primary hepatocytes of the mice of pAAV9-Con group and pAAV9-*Saas*-Sh group were isolated and cultured according to the procedures aforementioned. Hepatocyte supernatant was collected from the 12-well chambers seeded with 8 × 10^5^ murine primary hepatocytes in one chamber with 0.5 mL DMEM medium without fetal bovine serum for 12 h. Mouse SAAs enzyme-linked immunosorbent assay (ELISA) kit (#PS823, Beyotime, Shanghai, China) was applied to measure the concentration of DMEM medium control and hepatocyte supernatant.

### Survival analysis

The tissue samples of patients used for the survival analyses were split into two groups (high and low) according to the quantile expression of the proposed gene(s) in the surv_cutpoint function of the R survminer package. Kaplan-Meier survival curves measuring the fractions of patients living for a certain time were plotted to compare the two patient groups and assess the impact of the particular gene(s) on the prognosis. Statistical significance was calculated using the log-rank test. All analyses were performed using the R 3.6.0 framework.

### Statistical analysis

Statistical analyses were performed using the R 3.6.0 framework, including Student’s *t*-test, Wilcoxon’s sign rank test, and Wilcoxon’s rank-sum test. Asterisks represent the significance levels of the performed tests (*P < 0.05; **P < 0.01; ***P < 0.001).

## Supplementary information

Supplementary information Fig.S1

Supplementary information Fig. S2

Supplementary information Fig.S3

Supplementary information Fig.S4

Supplementary information Fig.S5

Supplementary information Fig.S6

Supplementary information Fig.S7

Supplementary information Fig.S8

Supplementary information Fig.S9

Supplementary information Fig.S10

Supplementary Table S1

Supplementary Table S2

Supplementary Table S3

Supplementary Table S4

Supplementary Table S5

## Data Availability

The data generated in this study have been filed in the China Human Genetic Resources Service Management System (Record Number: 2022BAT2001), which is open to the public. The Stereo-seq data and scRNA-seq data generated by this study have been deposited in the China National GeneBank (CNGB) Sequence Archive (CNSA, accession code: CNP0002199). The bulk RNA-seq and proteome data from validation cohort 5 (referenced in Supplementary information, Figs. S5b, S10f, g), are available from the National Genomics Data Center (NGDC) with accession number OEP000321.
