# Supplementary material for: An invasive zone in human liver cancer identified by Stereo-seq promotes hepatocyte–tumor cell crosstalk, local immunosuppression and tumor progression
Source: Cell Res. 2023 Jun 19;33(8):585–603. doi: 10.1038/s41422-023-00831-1 (PMC10397313; doi:10.1038/s41422-023-00831-1)
Supplement: Supplementary file 1 — Supplementary information Fig.S1 [file 41422_2023_831_MOESM1_ESM.pdf]

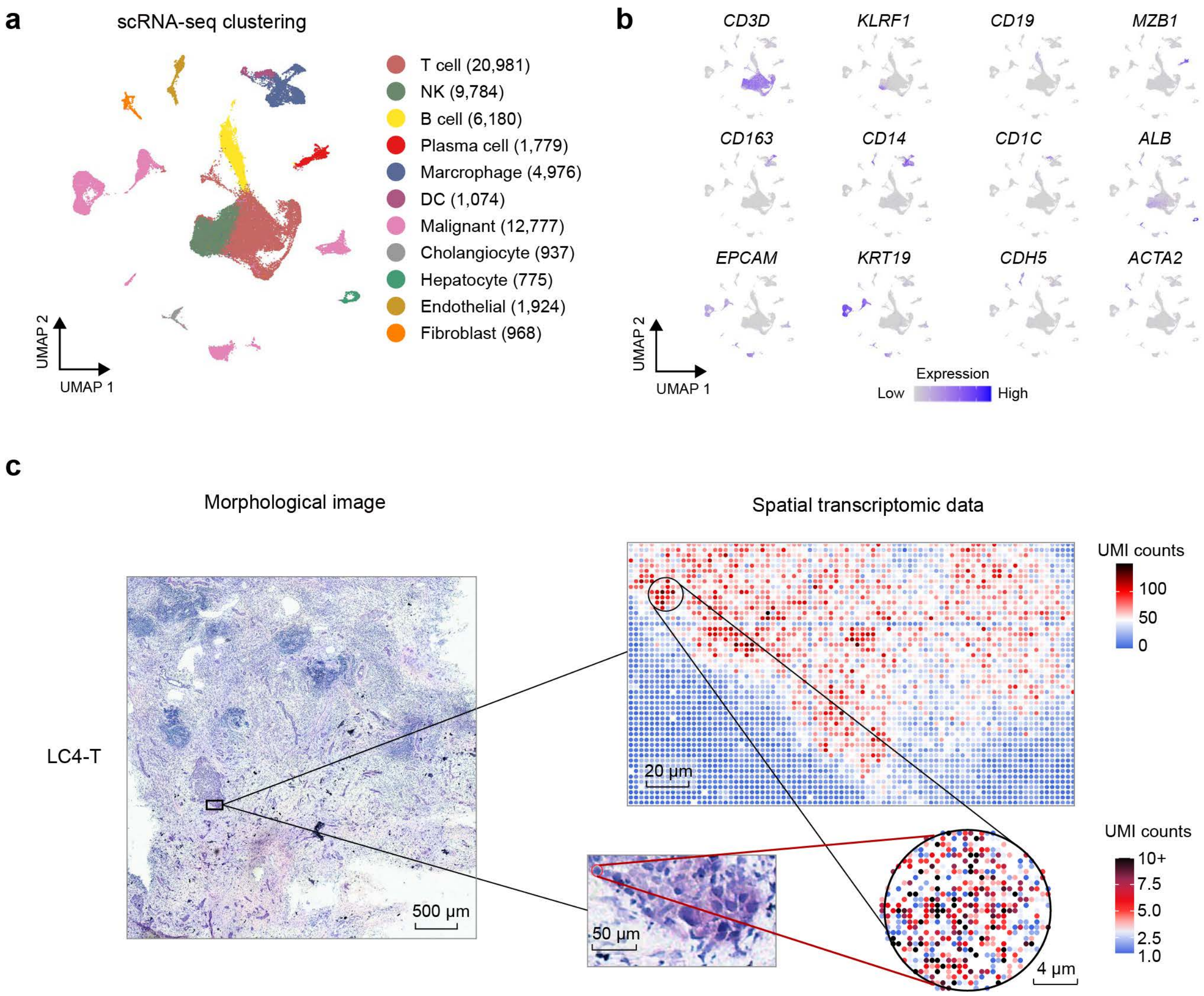

Supplementary information, Fig. S1. scRNA-seq profiling and spatial transcriptome profiling in liver cancer. a. A uniform manifold approximation and projection (UMAP) showing the classification of 62,155 single cells based on scRNA-seq data using Seurat. **b.** UMAP plots with color-coded marker genes indicating the expression levels (gray to purple) for major cell types. **c.** Stereo-seq spots were mapped to the H&E staining of adjacent slides on an LC4-T specimen, and the raw spatial expression matrix with each bin/spot was convoluted into 2.5 μm × 2.5 μm pseudo-spots (5 × 5 bins/spot) to show the unique molecular identifier (UMI) counts of the cell nucleus (with a diameter of ~15 μm) of a tumor cell.
