# Supplementary material for: An invasive zone in human liver cancer identified by Stereo-seq promotes hepatocyte–tumor cell crosstalk, local immunosuppression and tumor progression
Source: Cell Res. 2023 Jun 19;33(8):585–603. doi: 10.1038/s41422-023-00831-1 (PMC10397313; doi:10.1038/s41422-023-00831-1)
Supplement: Supplementary file 2 — Supplementary information Fig. S2 [file 41422_2023_831_MOESM2_ESM.pdf]

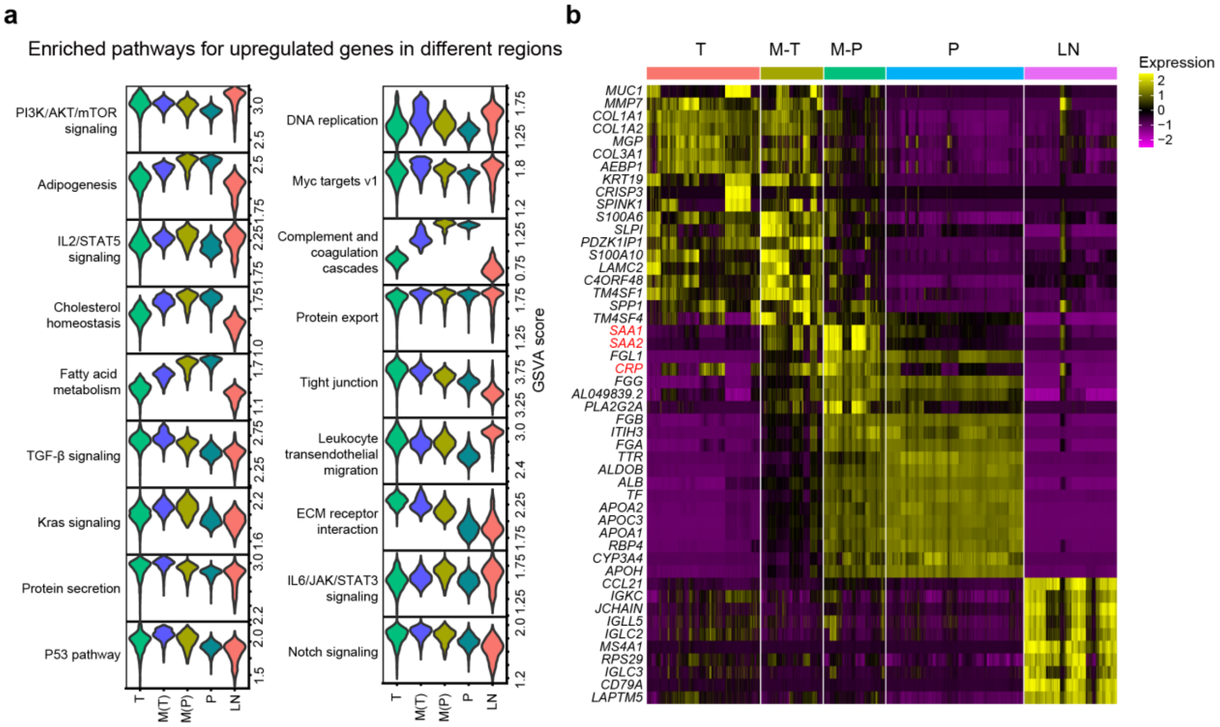

Supplementary information, Fig. S2. Pathway enrichment and differentially expressed genes (DEGs) in the four regions. a. Violin plots showing the GSVA enrichment scores of different pathways in four regions based on the Stereo-seq data from 21 patients with liver cancer. **b.** Heatmap representing the expression levels of DEGs among the four regional sites of 21 patients with liver cancer determined using the bin1000 based on Stereo-seq data. T, n = 12; M, n = 21; P, n = 10; LN, n = 10. Margin areas were further divided into the tumor part (M-T) and paratumor part (M-P) of margin areas according to the tumor border.
