# Supplementary material for: An invasive zone in human liver cancer identified by Stereo-seq promotes hepatocyte–tumor cell crosstalk, local immunosuppression and tumor progression
Source: Cell Res. 2023 Jun 19;33(8):585–603. doi: 10.1038/s41422-023-00831-1 (PMC10397313; doi:10.1038/s41422-023-00831-1)
Supplement: Supplementary file 3 — Supplementary information Fig.S3 [file 41422_2023_831_MOESM3_ESM.pdf]

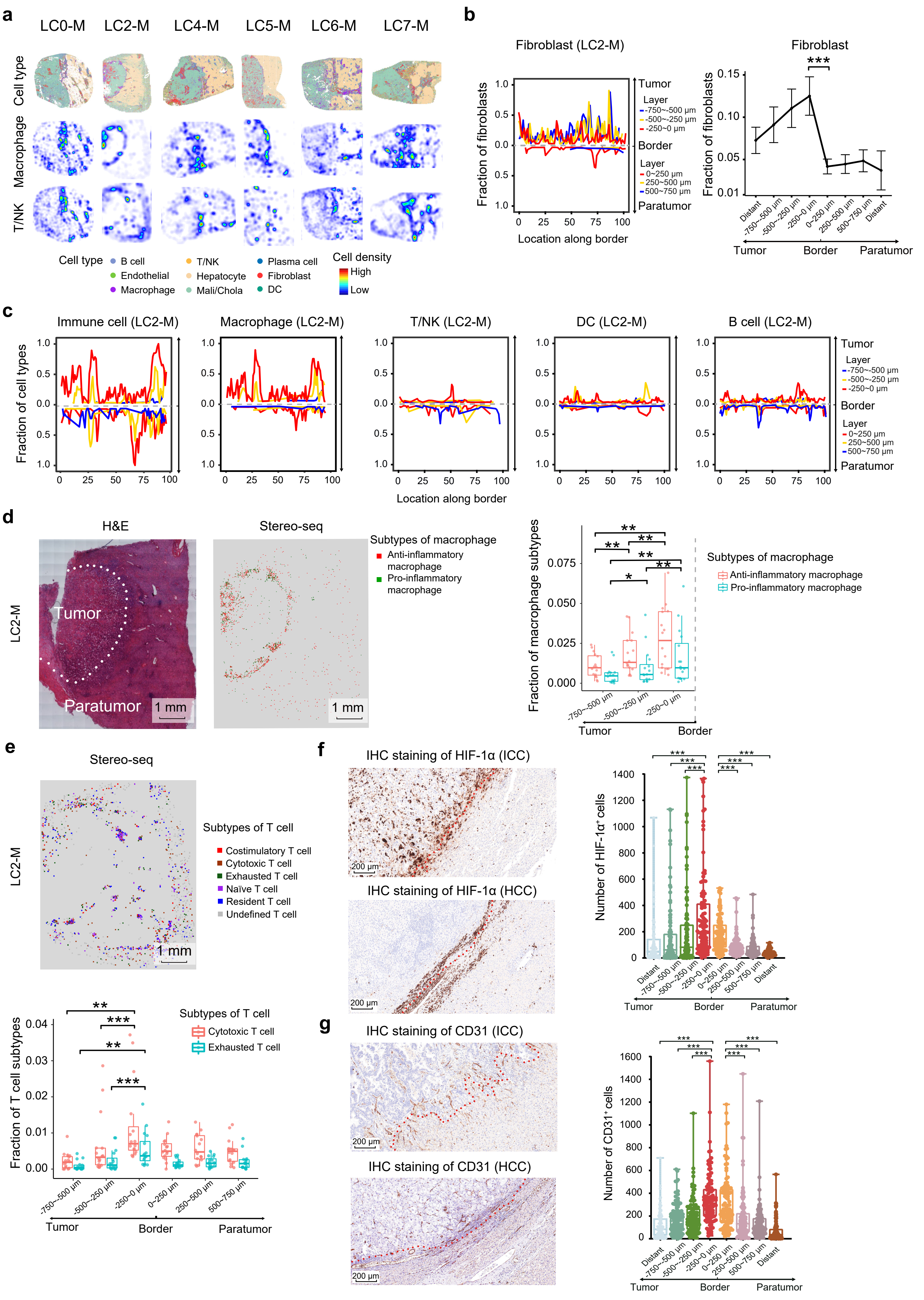

a
LC0-M LC2-M LC4-M LC5-M LC6-M LC7-M
Cell type
Macrophage
T/NK
Cell type: B cell, Endothelial, Macrophage, T/NK, Hepatocyte, Mali/Chola, Plasma cell, Fibroblast, DC
Cell density: High, Low
b
Fibroblast (LC2-M)
Fraction of fibroblasts
Location along border
Tumor
Layer
-750~-500 μm
-500~-250 μm
-250~0 μm
Border
Layer
0~250 μm
250~500 μm
500~750 μm
Paratumor
Fibroblast
***
Distant, -750~-500 μm, -500~-250 μm, -250~0 μm, 0~250 μm, 250~500 μm, 500~750 μm, Distant
Tumor Border Paratumor
c
Immune cell (LC2-M)
Macrophage (LC2-M)
T/NK (LC2-M)
DC (LC2-M)
B cell (LC2-M)
Fraction of cell types
Location along border
d
H&E
Stereo-seq
LC2-M
Tumor
Paratumor
1 mm
Subtypes of macrophage
Anti-inflammatory macrophage
Pro-inflammatory macrophage
Fraction of macrophage subtypes
Tumor Border
e
Stereo-seq
LC2-M
1 mm
Subtypes of T cell
Costimulatory T cell
Cytotoxic T cell
Exhausted T cell
Naïve T cell
Resident T cell
Undefined T cell
Fraction of T cell subtypes
Tumor Border Paratumor
f
IHC staining of HIF-1α (ICC)
IHC staining of HIF-1α (HCC)
200 μm
Number of HIF-1α+ cells
Tumor Border Paratumor
g
IHC staining of CD31 (ICC)
IHC staining of CD31 (HCC)
200 μm
Number of CD31+ cells
Tumor Border Paratumor

Supplementary information, Fig. S3. The spatial distribution of immune cells in margin areas. a. Spatial distribution maps of all cell types and spatial heatmaps of macrophages and T/NK cells in margin areas. **b.** Line graph showing the fractions of fibroblasts in all cell components in the tangential direction along the border (100 subregions for each layer) of three layers from the paratumor and tumor sides (LC2-M, left panel), and a box plot showing the fractions of fibroblasts in all cell components in the normal direction of different layers moving away from the border (right panel) as determined from the Stereo-seq data of 16 patients with liver cancer. **c.** Line graphs showing the fractions of immune cells, macrophages, T/NK cells, DC cells, and B cells in all cell components along the border (100 subregions for each layer) of three layers from the paratumor and tumor sides (LC2-M). **d.** H&E staining and the corresponding spatial distribution maps of pro-inflammatory and anti-inflammatory macrophages (LC2-M) of adjacent ST slide and box plots showing pro-inflammatory and anti-inflammatory macrophage ratios in immune cells in different layers from the tumor side (n = 16). **e.** Spatial distribution maps of T cell subtypes and box plots showing the fractions of cytotoxic T cells and exhausted T cells in all cell components in different layers of margin areas (n = 16). **(f-g)** IHC staining images and quantification data of HIF-1α$^+$ cells **(f)** and CD31$^+$ cells **(g)** number in the invasion zone (1000 μm in normal length) of Validation Cohort 1 including 53 HCC patients and 52 ICC patients. Student's t-test was used to analyze the data shown in panels **b, d, e, f** and **g**. *, represents $P < 0.05$; **, represents $P < 0.01$; ***, represents $P < 0.001$. Mali/Chola, malignant cells or cholangiocytes.
