# Supplementary material for: An invasive zone in human liver cancer identified by Stereo-seq promotes hepatocyte–tumor cell crosstalk, local immunosuppression and tumor progression
Source: Cell Res. 2023 Jun 19;33(8):585–603. doi: 10.1038/s41422-023-00831-1 (PMC10397313; doi:10.1038/s41422-023-00831-1)
Supplement: Supplementary file 4 — Supplementary information Fig.S4 [file 41422_2023_831_MOESM4_ESM.pdf]

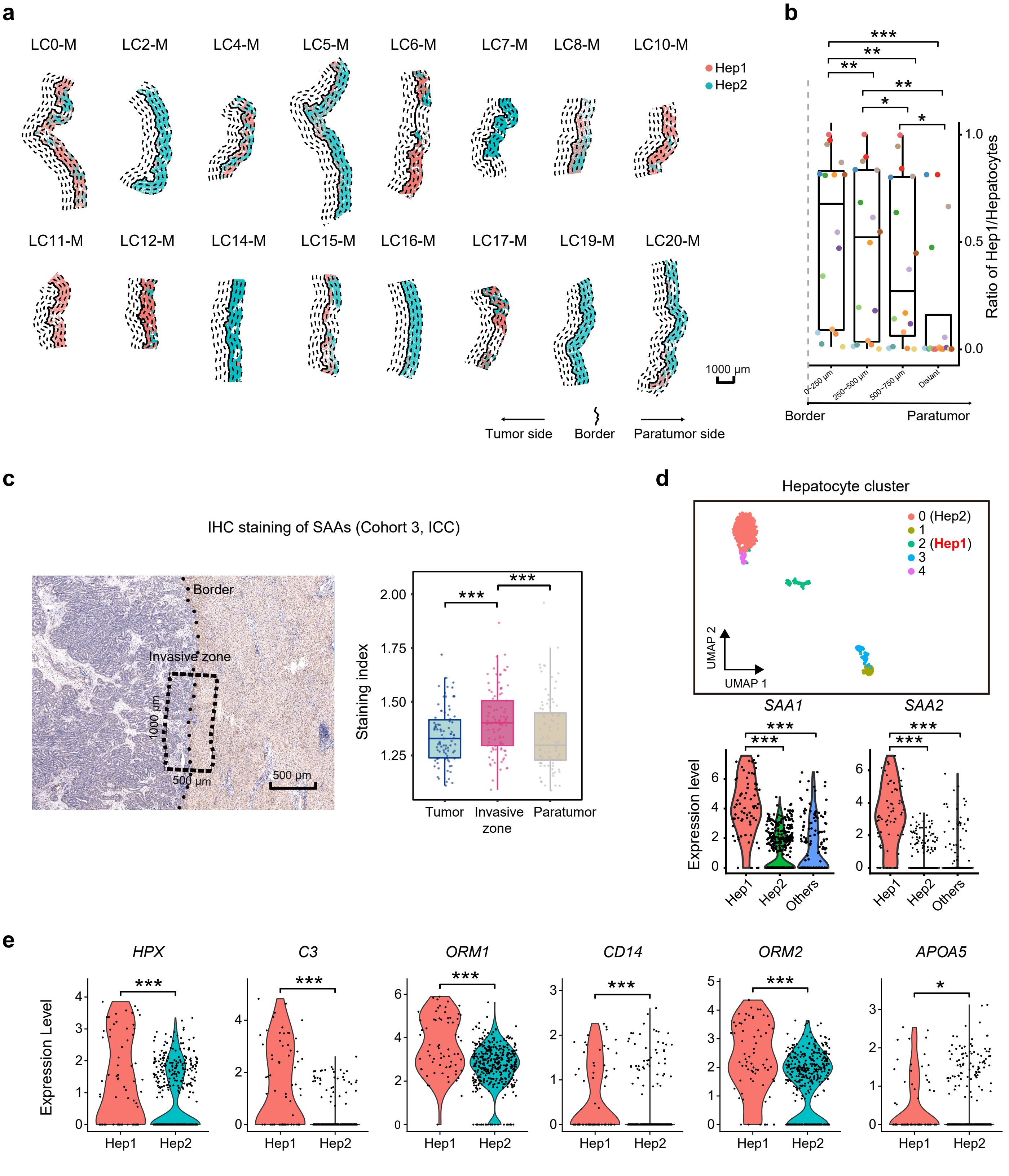

a
LC0-M
LC2-M
LC4-M
LC5-M
LC6-M
LC7-M
LC8-M
LC10-M
Hep1
Hep2
LC11-M
LC12-M
LC14-M
LC15-M
LC16-M
LC17-M
LC19-M
LC20-M
1000 μm
Tumor side
Border
Paratumor side
b
Ratio of Hep1/Hepatocytes
Border
Paratumor
Distant
c
IHC staining of SAAs (Cohort 3, ICC)
Border
Invasive zone
1000 μm
500 μm
500 μm
Staining index
Tumor
Invasive zone
Paratumor
d
Hepatocyte cluster
0 (Hep2)
1
2 (Hep1)
3
4
UMAP 2
UMAP 1
SAA1
SAA2
Expression level
Hep1
Hep2
Others
e
HPX
C3
ORM1
CD14
ORM2
APOA5
Expression Level
Hep1
Hep2

Supplementary information, Fig. S4. The expression of SAAs is upregulated in hepatocytes in the invasive zone. a. Spatial distribution maps of Hep1 and Hep2 among different layers from the tumor side of the border in Stereo-seq slides (n =16). **b.** Box plot of the ratios of Hep1 to all hepatocytes in different layers from the paratumor side of the border (16 patients). **c.** Diagram of the sampling areas (500 μm × 1,000 μm zone) of the invasive zone used for the IHC staining of SAAs in the ICC samples and for the quantitative analysis of the IHC staining index of the SAAs for 93 ICC patients from Validation Cohort 3. The zones from tumor or paratumor tissues were acquired from the areas at least 1 mm from the border, and three different repeated zones were used to acquire an average value for the staining index. **d.** UMAP plot for hepatocyte cluster identification (upper panel) and violin plots (lower panel) representing the *SAA1* and *SAA2* expression levels among different clusters based on scRNA-seq data. **e.** Violin plots representing the expression levels of genes encoding representative acute-phase proteins including *HPX, C3, CD14, ORM1, ORM2,* and *APOA5* in Hep1 and Hep2 subtypes based on scRNA-seq data. The paired Student's t-test was used to analyze the data in panels **b-c**, and the Wilcoxon test was used in panels **d-e**. *, represents $P < 0.05$; **, represents $P < 0.01$; ***, represents $P < 0.001$.
