# Supplementary material for: An invasive zone in human liver cancer identified by Stereo-seq promotes hepatocyte–tumor cell crosstalk, local immunosuppression and tumor progression
Source: Cell Res. 2023 Jun 19;33(8):585–603. doi: 10.1038/s41422-023-00831-1 (PMC10397313; doi:10.1038/s41422-023-00831-1)
Supplement: Supplementary file 5 — Supplementary information Fig.S5 [file 41422_2023_831_MOESM5_ESM.pdf]

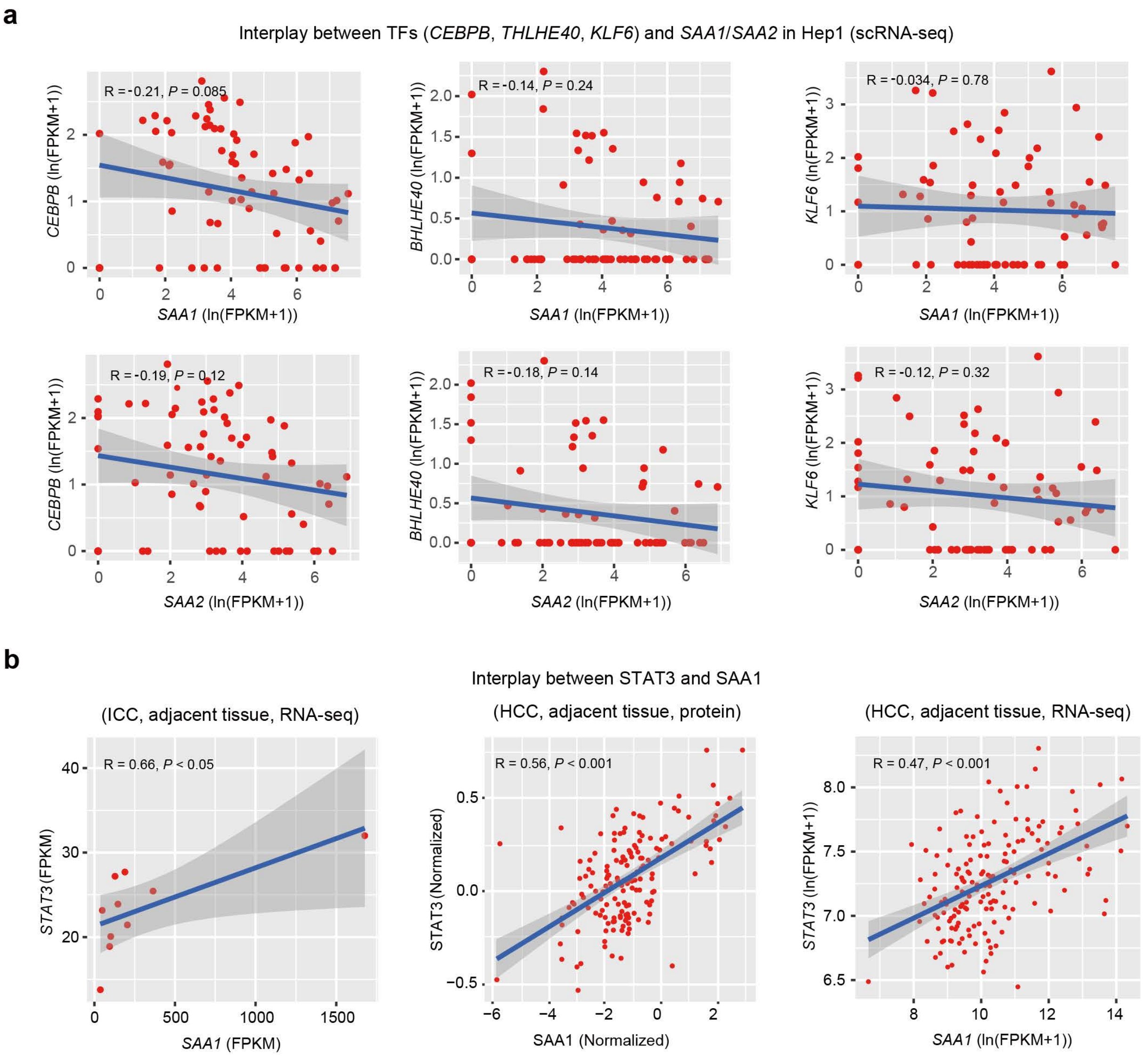

Supplementary information, Fig. S5. JAK-STAT3 activation is associated with high SAAs expression of hepatocytes in the invasive zone. a. Scatter plots showing the correlation between the expression levels of genes encoding transcription factors (including *CEBPB*, *THLHE40*, *KLF*6) and *SAA1*/*SAA2* in Hep1 based on the scRNA-seq data. **b.** Scatter plots showing positive correlations between STAT3 and SAA1 expression in adjacent tissues (ICC, Cohort 2, n = 10; HCC, Cohort 5, n = 159).
