# Supplementary material for: An invasive zone in human liver cancer identified by Stereo-seq promotes hepatocyte–tumor cell crosstalk, local immunosuppression and tumor progression
Source: Cell Res. 2023 Jun 19;33(8):585–603. doi: 10.1038/s41422-023-00831-1 (PMC10397313; doi:10.1038/s41422-023-00831-1)
Supplement: Supplementary file 6 — Supplementary information Fig.S6 [file 41422_2023_831_MOESM6_ESM.pdf]

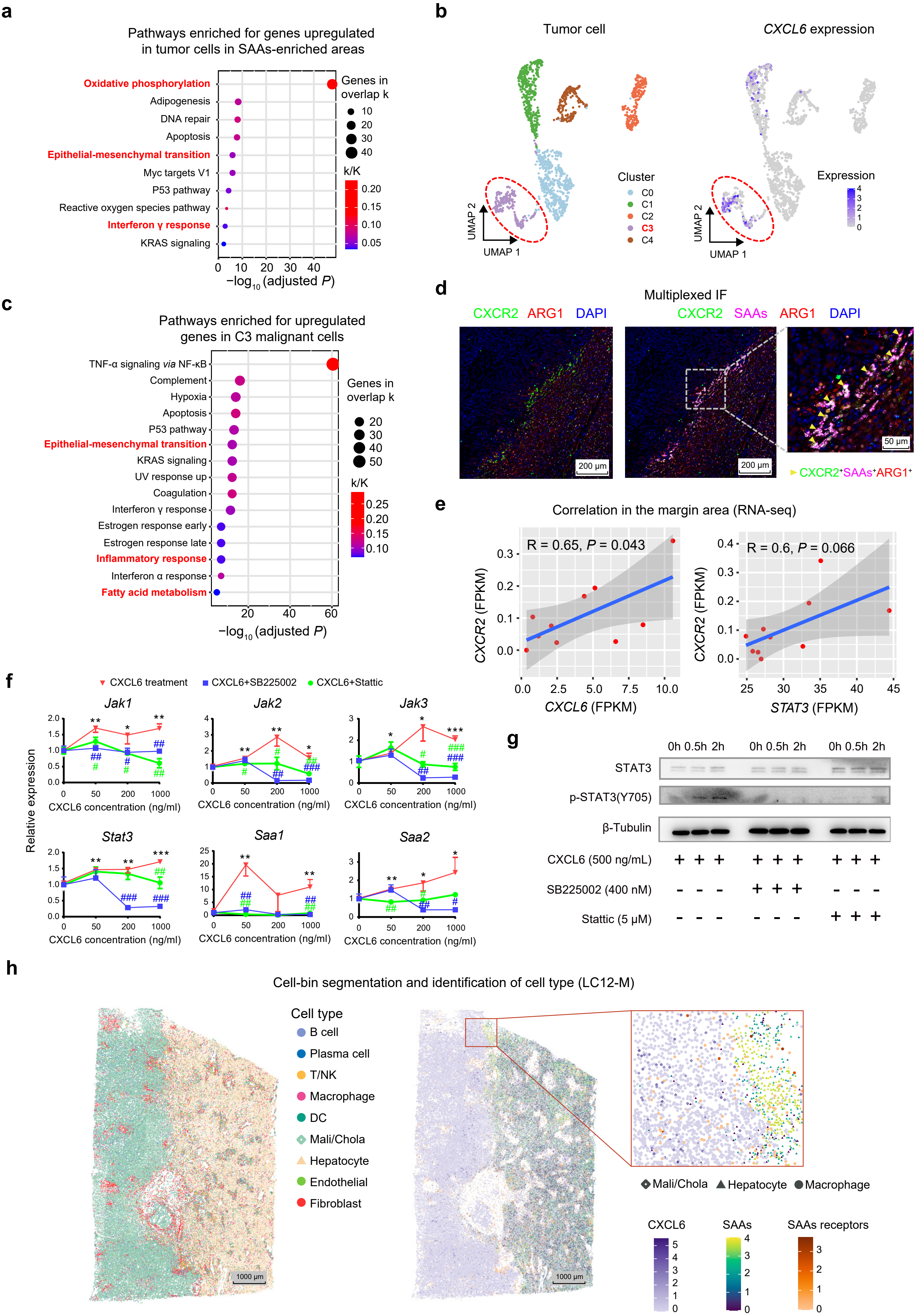

a
Pathways enriched for genes upregulated in tumor cells in SAAs-enriched areas
Oxidative phosphorylation
Adipogenesis
DNA repair
Apoptosis
Epithelial-mesenchymal transition
Myc targets V1
P53 pathway
Reactive oxygen species pathway
Interferon γ response
KRAS signaling
Genes in overlap k
k/K
−log10 (adjusted P)
b
Tumor cell
CXCL6 expression
Cluster
C0 C1 C2 C3 C4
Expression
UMAP 1
UMAP 2
c
Pathways enriched for upregulated genes in C3 malignant cells
TNF-α signaling via NF-κB
Complement
Hypoxia
Apoptosis
P53 pathway
Epithelial-mesenchymal transition
KRAS signaling
UV response up
Coagulation
Interferon γ response
Estrogen response early
Estrogen response late
Inflammatory response
Interferon α response
Fatty acid metabolism
Genes in overlap k
k/K
−log10 (adjusted P)
d
Multiplexed IF
CXCR2 ARG1 DAPI
CXCR2 SAAs ARG1 DAPI
200 μm
50 μm
CXCR2+SAAs+ARG1+
e
Correlation in the margin area (RNA-seq)
R = 0.65, P = 0.043
R = 0.6, P = 0.066
CXCR2 (FPKM)
CXCL6 (FPKM)
STAT3 (FPKM)
f
CXCL6 treatment
CXCL6+SB225002
CXCL6+Stattic
Jak1
Jak2
Jak3
Stat3
Saa1
Saa2
Relative expression
CXCL6 concentration (ng/ml)
g
0h 0.5h 2h
STAT3
p-STAT3(Y705)
β-Tubulin
CXCL6 (500 ng/mL)
SB225002 (400 nM)
Stattic (5 μM)
h
Cell-bin segmentation and identification of cell type (LC12-M)
Cell type
B cell
Plasma cell
T/NK
Macrophage
DC
Mali/Chola
Hepatocyte
Endothelial
Fibroblast
1000 μm
Mali/Chola
Hepatocyte
Macrophage
CXCL6
SAAs
SAAs receptors

Supplementary information, Fig. S6. Identification of CXCL6^{+} tumor cells contributing to the high SAAs expression of hepatocytes in the invasive zone. a. A pathway enrichment analysis using hallmark gene sets for genes upregulated in tumor cells in the SAAs-enriched areas of the invasive zone, when compared with those in remnant regions of the invasive zone. k is the number of genes in the intersection of the query set with a set from database and K is the number of genes in the set from database. **b.** UMAP plots showing tumor cell cluster identification and *CXCL6* expression in different clusters based on the scRNA-seq data. **c.** Enriched pathways in hallmark gene sets for upregulated genes of the C3 cluster of tumor cells based on scRNA-seq. k is the number of genes in the intersection of the query set with a set from database and K is the number of genes in the set from database. **d.** Multiplexed IF staining (ARG1, CXCR2, SAAs, and DAPI) showing high expression of CXCR2 specifically in SAAs^{+} hepatocytes in the invasive zone (the ICC patient from Validation Cohort 4). **e.** Scatter plot illustrating the correlations between the expression levels of *CXCL6* or *STAT3* and *CXCR2* in the margin areas of specimens from 10 ICC patients from Validation Cohort 2 determined using bulk RNA-seq. **f.** Relative transcriptional expression of JAK-STAT3 related genes in murine primary hepatocytes treated by mouse recombinant GCP2/CXCL6 protein (0 ng/ml, 20 ng/ml, 200 ng/ml, and 1000 ng/ml) with or without CXCR2 inhibitor (SB225002) or STAT3 inhibitor (Stattic) for 12 h. **g.** Western blot images of STAT3 activation by phosphorylation of STAT3 (Y705) at indicated time points after CXCL6 treatment with or without inhibition of CXCR2 or STAT3. **h.** Spatial identification of the local ecosystem along the border and identification of SAAs^{+} hepatocytes, CXCL6^{+} tumor cells, and SAAs receptor^{+} macrophages in the Stereo-seq slide (LC12-M) using cell-bin at the single-cell resolution. Student's *t*-test was used to analyze the data in Panel **f**. *, represents $P < 0.05$; **, represents $P < 0.01$; ***, represents $P < 0.001$. Mali/Chola, malignant cells or cholangiocytes.
