# Supplementary material for: An invasive zone in human liver cancer identified by Stereo-seq promotes hepatocyte–tumor cell crosstalk, local immunosuppression and tumor progression
Source: Cell Res. 2023 Jun 19;33(8):585–603. doi: 10.1038/s41422-023-00831-1 (PMC10397313; doi:10.1038/s41422-023-00831-1)
Supplement: Supplementary file 7 — Supplementary information Fig.S7 [file 41422_2023_831_MOESM7_ESM.pdf]

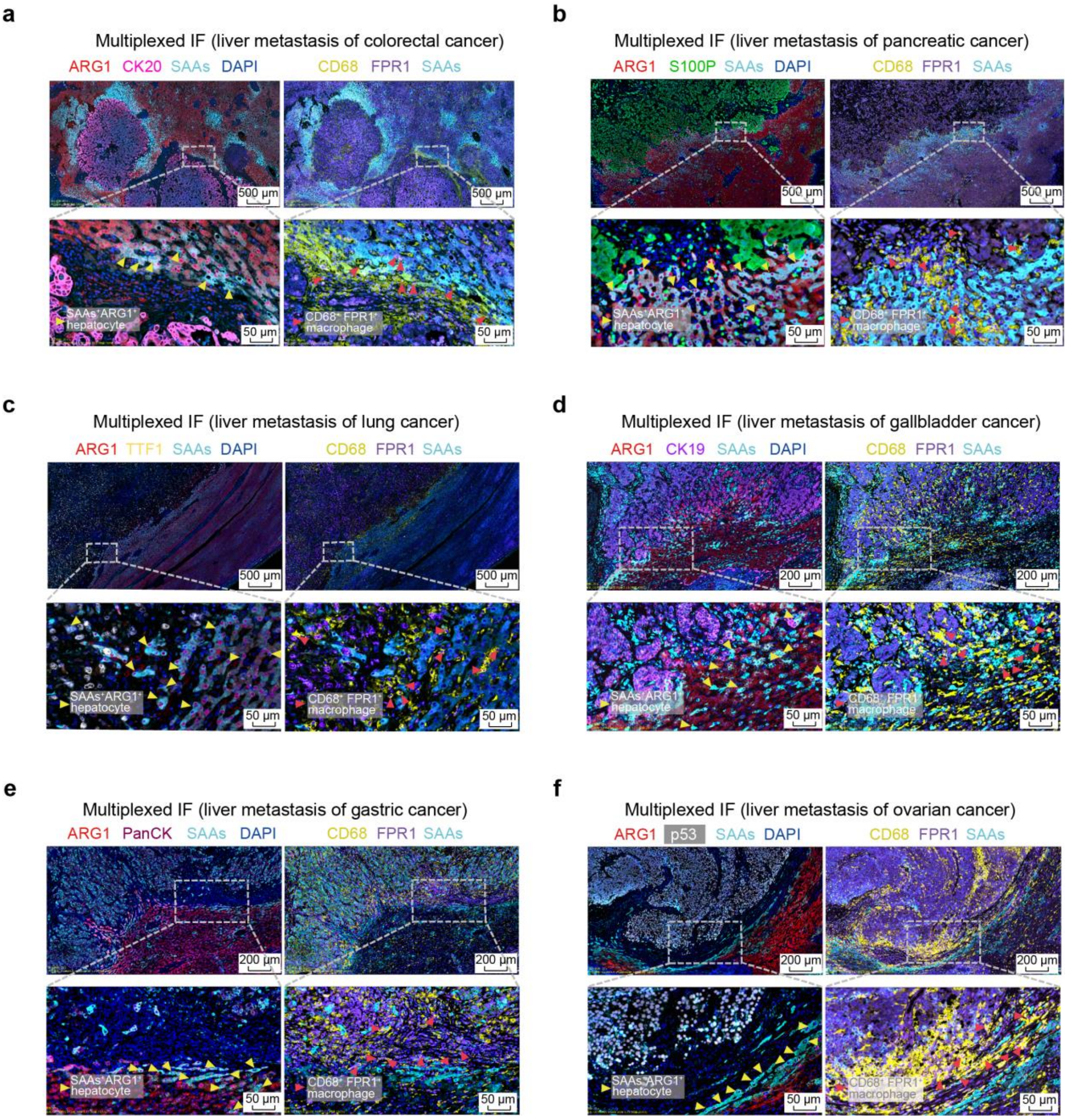

Supplementary information, Fig. S7. Accumulation of FPR1$^+$ macrophages recruited by SAAs secreted from damaged hepatocytes in the invasive zone of liver metastasis from different cancer types. a. Multiplexed IF staining (ARG1, CK20, FPR1, CD68, SAAs, and DAPI) showing co-aggregation of FPR1$^+$ macrophages (FPR1$^+$CD68$^+$ cells) and SAAs$^+$ hepatocytes (SAAs$^+$ARG1$^+$ cells) in the invasive zone of a specimen from a patient with liver metastasis of colorectal cancer. **b.** Multiplexed IF staining (ARG1, S100P, FPR1, CD68, SAAs, and DAPI) of FPR1$^+$ macrophages (FPR1$^+$CD68$^+$ cells) and SAAs$^+$ hepatocytes (SAAs$^+$ARG1$^+$ cells) in the invasive zone of a specimen from a patient with liver metastasis of pancreatic cancer. **c.** Multiplexed IF staining (ARG1, TTF1, FPR1, CD68, SAAs, and DAPI) of FPR1$^+$ macrophages (FPR1$^+$CD68$^+$ cells) and SAAs$^+$ hepatocytes (SAAs$^+$ARG1$^+$ cells) in the invasive zone of a specimen from a patient with liver metastasis of lung cancer. **d.** Multiplexed IF staining (ARG1, CK19, FPR1, CD68, SAAs, and DAPI) of FPR1$^+$ macrophages (FPR1$^+$CD68$^+$ cells) and SAAs$^+$ hepatocytes (SAAs$^+$ARG1$^+$ cells) in the invasive

zone of a specimen from a patient with liver metastasis of gallbladder cancer. **e.** Multiplexed IF staining (ARG1, PanCK, FPR1, CD68, SAAs, and DAPI) of $FPR1^+$ macrophages ($FPR1^+CD68^+$ cells) and $SAAs^+$ hepatocytes ($SAAs^+ARG1^+$ cells) in the invasive zone of a specimen from a patient with liver metastasis of gastric cancer. **f.** Multiplexed IF staining (ARG1, p53, FPR1, CD68, SAAs, and DAPI) of $FPR1^+$ macrophages ($FPR1^+CD68^+$ cells) and $SAAs^+$ hepatocytes ($SAAs^+ARG1^+$ cells) in the invasive zone of a specimen from a patient with liver metastasis of ovarian cancer.
