# Supplementary material for: An invasive zone in human liver cancer identified by Stereo-seq promotes hepatocyte–tumor cell crosstalk, local immunosuppression and tumor progression
Source: Cell Res. 2023 Jun 19;33(8):585–603. doi: 10.1038/s41422-023-00831-1 (PMC10397313; doi:10.1038/s41422-023-00831-1)
Supplement: Supplementary file 8 — Supplementary information Fig.S8 [file 41422_2023_831_MOESM8_ESM.pdf]

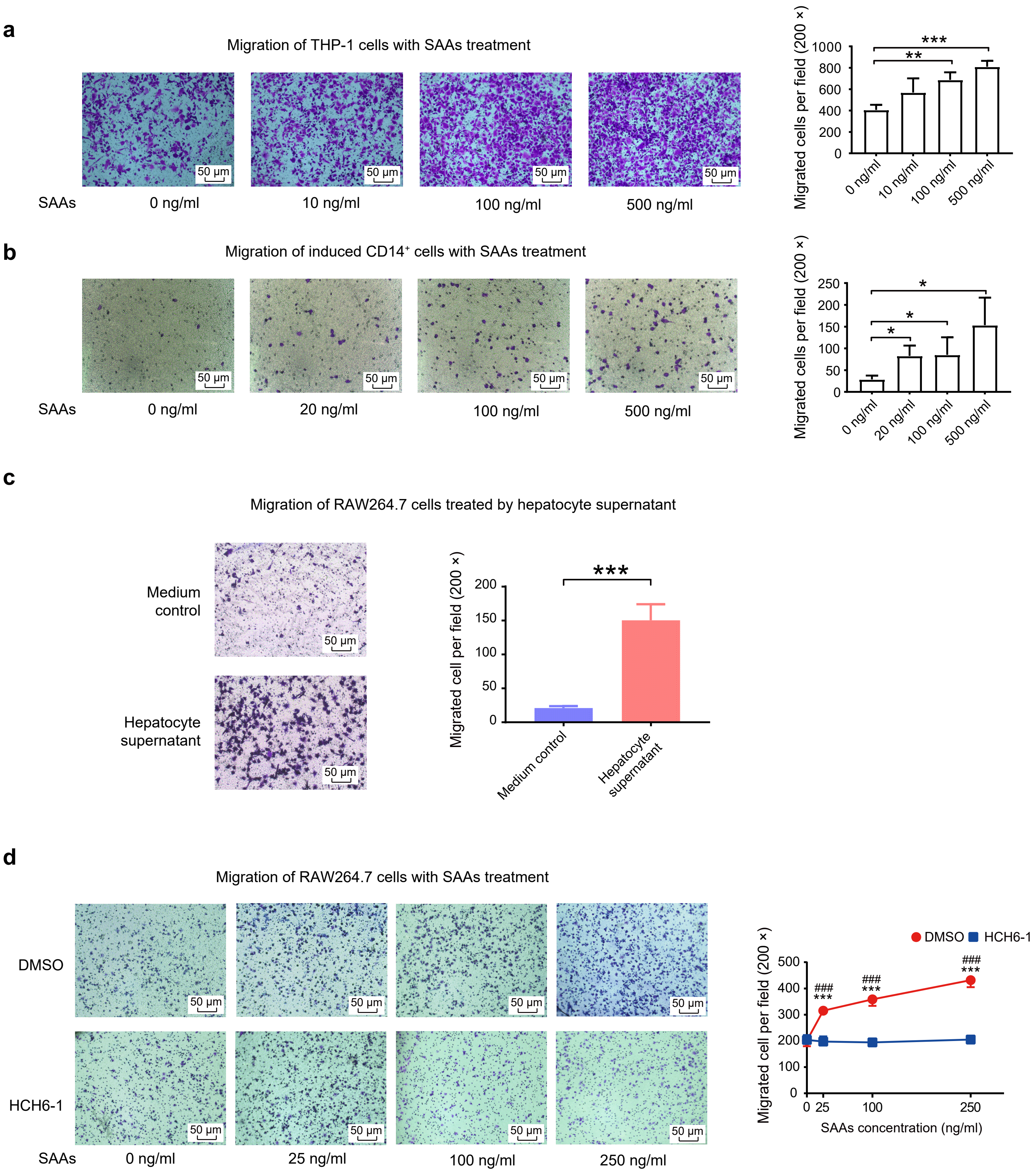
a
Migration of THP-1 cells with SAAs treatment
50 μm
SAAs
0 ng/ml
10 ng/ml
100 ng/ml
500 ng/ml
Migrated cells per field (200 ×)
1000
800
600
400
200
0
***
**
0 ng/ml
10 ng/ml
100 ng/ml
500 ng/ml
b
Migration of induced CD14+ cells with SAAs treatment
50 μm
SAAs
0 ng/ml
20 ng/ml
100 ng/ml
500 ng/ml
Migrated cells per field (200 ×)
250
200
150
100
50
0
*
*
*
0 ng/ml
20 ng/ml
100 ng/ml
500 ng/ml
c
Migration of RAW264.7 cells treated by hepatocyte supernatant
Medium control
Hepatocyte supernatant
50 μm
Migrated cell per field (200 ×)
200
150
100
50
0
***
Medium control
Hepatocyte supernatant
d
Migration of RAW264.7 cells with SAAs treatment
DMSO
HCH6-1
50 μm
SAAs
0 ng/ml
25 ng/ml
100 ng/ml
250 ng/ml
DMSO
HCH6-1
Migrated cell per field (200 ×)
500
400
300
200
100
0
###
***
0
25
100
250
SAAs concentration (ng/ml)

Supplementary information, Fig. S8. Migration of macrophages induced by SAAs. a. Representative images (left panel) and cell number quantification (right panel) in a 200 × field for a migration assay of THP-1 cells treated with SAAs (0 ng/ml, 10 ng/ml, 100 ng/ml, and 500 ng/ml) for two days. **b.** Representative images (left panel) and migrated cells number quantification (right panel) in a 200 × field for a migration assay of $CD14^+$ PBMCs treated with SAAs (0 ng/ml, 20 ng/ml, 100 ng/ml, and 500 ng/ml) for two days. **c.** Representative images (left panel) and migrated cells number quantification (right panel) in a 200 × field for RAW264.7 cells treated with medium control without foetal bovine serum or hepatocyte supernatant for 24 hours. **d.** Representative images (left panel) and cell number quantification (right panel) in a 200 × field for a migration assay of RAW264.7 cells treated with SAAs (0 ng/ml, 25 ng/ml, 100 ng/ml, and 250 ng/ml) with or without HCH6-1 (FPR1 inhibitor, 3 μM) in the lower compartments for 24 hours. * indicates the comparison to 0 ng/ml SAAs treatment; $^{\#}$ indicates the comparison to the HCH6-1 treatment group. Student's *t*-test was used to analyze the data in Panels **a**, **b**, **c, and d**. *, represents $P < 0.05$; **, represents $P < 0.01$; *** or $^{\#\#\#}$, represents $P < 0.001$.
