# Supplementary material for: An invasive zone in human liver cancer identified by Stereo-seq promotes hepatocyte–tumor cell crosstalk, local immunosuppression and tumor progression
Source: Cell Res. 2023 Jun 19;33(8):585–603. doi: 10.1038/s41422-023-00831-1 (PMC10397313; doi:10.1038/s41422-023-00831-1)
Supplement: Supplementary file 9 — Supplementary information Fig.S9 [file 41422_2023_831_MOESM9_ESM.pdf]

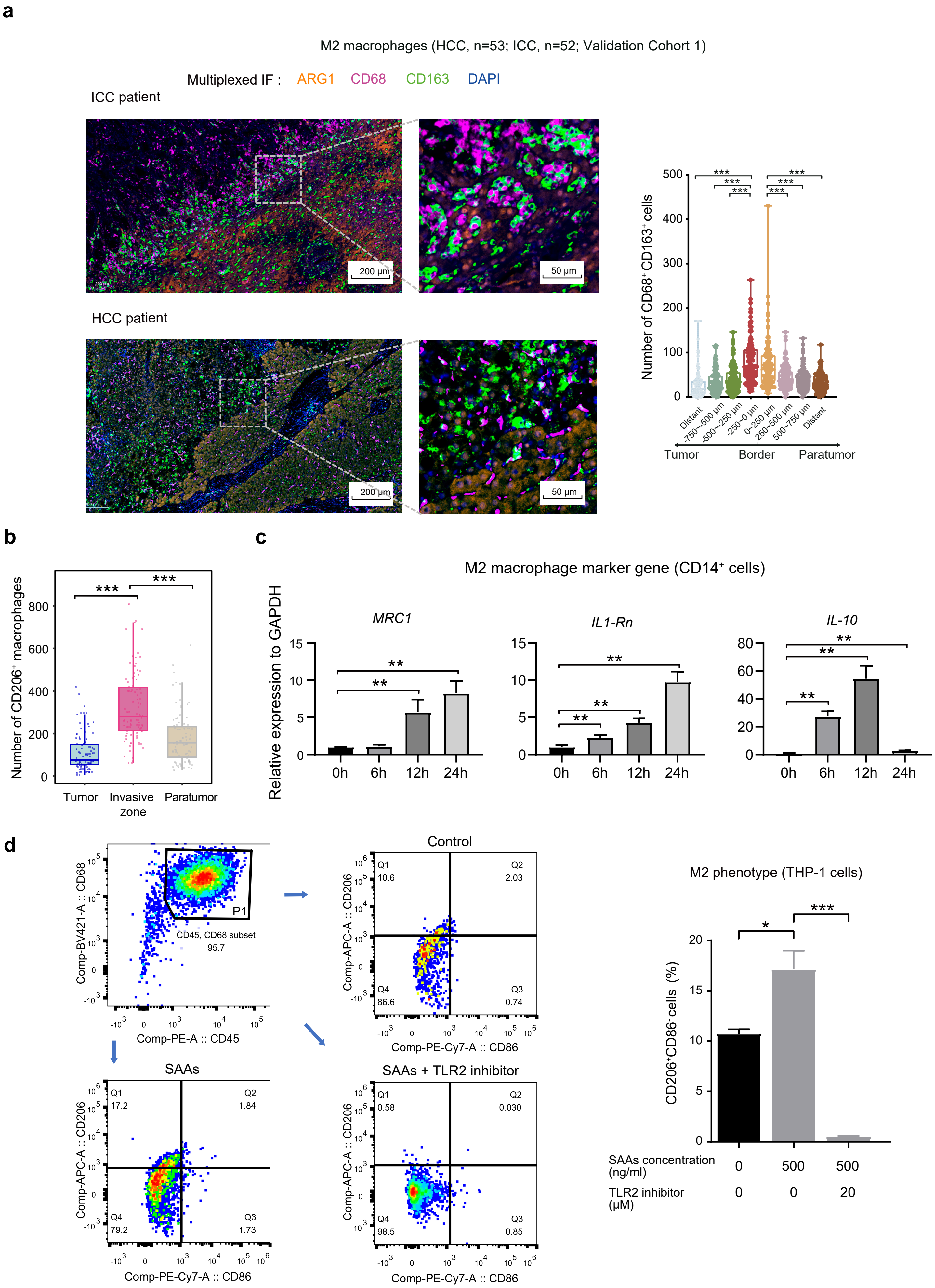

a
M2 macrophages (HCC, n=53; ICC, n=52; Validation Cohort 1)
Multiplexed IF : ARG1 CD68 CD163 DAPI
ICC patient
200 μm
50 μm
HCC patient
200 μm
50 μm
Number of CD68⁺ CD163⁺ cells
Distant
-750~-500 μm
-500~-250 μm
-250~0 μm
0~250 μm
250~500 μm
500~750 μm
Distant
Tumor
Border
Paratumor
b
Number of CD206⁺ macrophages
Tumor
Invasive zone
Paratumor
c
M2 macrophage marker gene (CD14⁺ cells)
Relative expression to GAPDH
MRC1
IL1-Rn
IL-10
0h
6h
12h
24h
d
Comp-BV421-A :: CD68
Comp-PE-A :: CD45
P1
CD45, CD68 subset
95.7
Control
Comp-APC-A :: CD206
Comp-PE-Cy7-A :: CD86
Q1 10.6
Q2 2.03
Q4 86.6
Q3 0.74
SAAs
Q1 17.2
Q2 1.84
Q4 79.2
Q3 1.73
SAAs + TLR2 inhibitor
Q1 0.58
Q2 0.030
Q4 98.5
Q3 0.85
M2 phenotype (THP-1 cells)
CD206⁺CD86⁻ cells (%)
SAAs concentration (ng/ml) 0 500 500
TLR2 inhibitor (μM) 0 0 20

Supplementary information, Fig. S9. Pro-tumor M2 phenotype of macrophages induced by SAAs. a. Multiplexed IF staining (CD68, CD163, ARG1, and DAPI) images and quantification of M2 macrophages ($CD68^+CD163^+$ cells) of different layers (1000 μm in normal length) in margin areas of primary liver cancer patients (HCC, n=53; ICC, n=52; Validation Cohort 1). **b.** Quantitative analysis of the IHC staining index for CD206 in the three zones of 93 ICC patients from Validation Cohort 3. Zones from tumor or paratumor tissues were acquired from the areas at least 1 mm from the border, and the average numbers of $CD206^+$ cells in three different areas from each zone were calculated. **c.** The relative transcriptional expression of M2 macrophage marker genes including *MRC1*, *IL1-Rn*, and *IL-10* in selected $CD14^+$ PBMCs from human whole blood cells treated with SAAs (200 ng/ml) in different time points. **d.** Flow cytometry showing the percentage of M2 macrophages ($CD68^+CD206^+CD86^-$ cells) in macrophage (P1, marked as $CD45^+CD68^+$ cells) treated with SAAs or SAAs plus TLR2 inhibitor. Student's *t*-test was used to analyze the data. * represents $P < 0.05$; **, represents $P < 0.01$; ***, represents $P < 0.001$.
