# Supplementary material for: An invasive zone in human liver cancer identified by Stereo-seq promotes hepatocyte–tumor cell crosstalk, local immunosuppression and tumor progression
Source: Cell Res. 2023 Jun 19;33(8):585–603. doi: 10.1038/s41422-023-00831-1 (PMC10397313; doi:10.1038/s41422-023-00831-1)
Supplement: Supplementary file 10 — Supplementary information Fig.S10 [file 41422_2023_831_MOESM10_ESM.pdf]

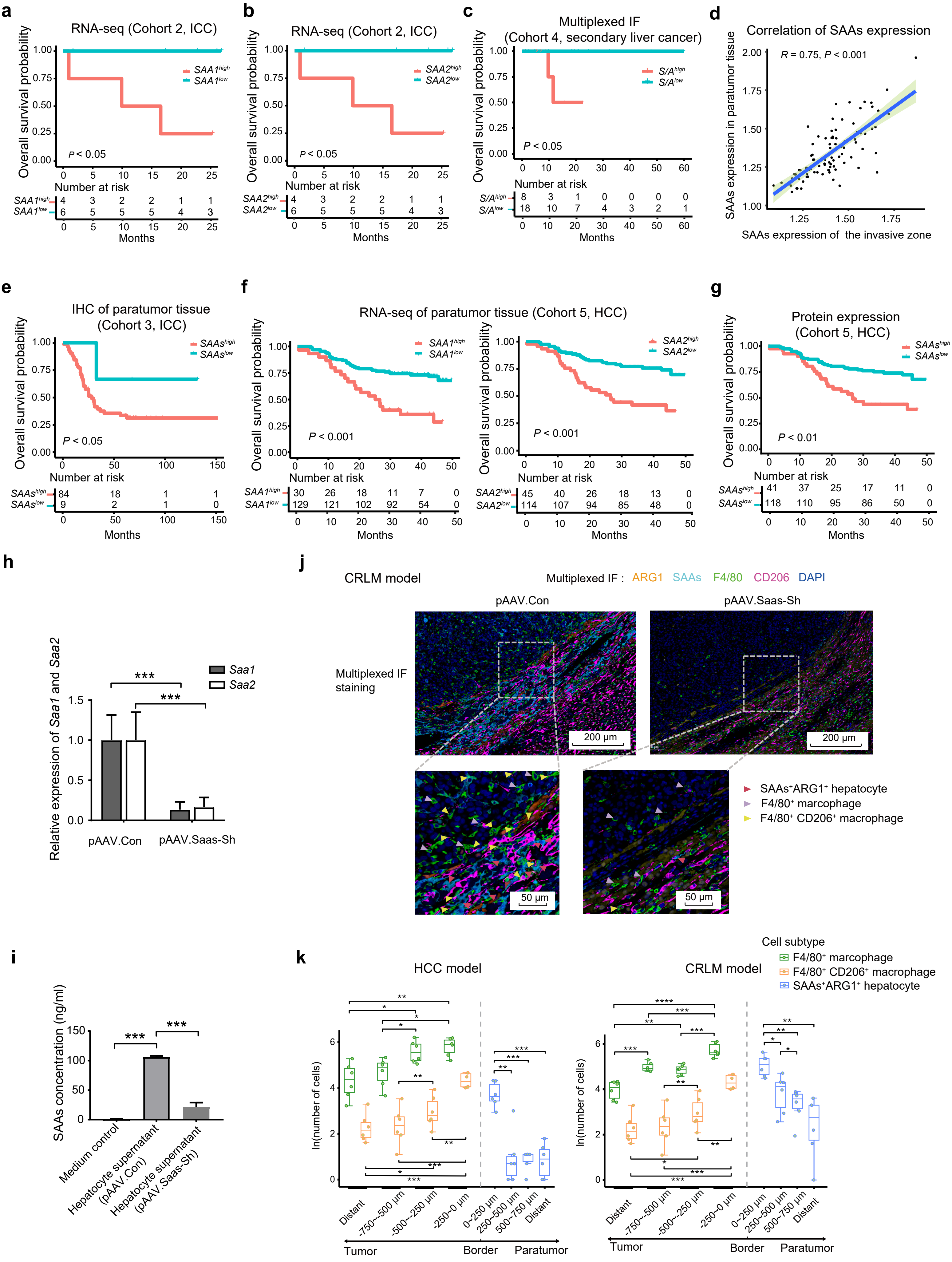
a
RNA-seq (Cohort 2, ICC)
Overall survival probability
SAA1high
SAA1low
P < 0.05
Number at risk
SAA1high 4 3 2 2 1 1
SAA1low 6 5 5 5 4 3
Months
b
RNA-seq (Cohort 2, ICC)
SAA2high
SAA2low
P < 0.05
Number at risk
SAA2high 4 3 2 2 1 1
SAA2low 6 5 5 5 4 3
Months
c
Multiplexed IF
(Cohort 4, secondary liver cancer)
S/Ahigh
S/Alow
P < 0.05
Number at risk
S/Ahigh 8 3 1 0 0 0 0
S/Alow 18 10 7 4 3 2 1
Months
d
Correlation of SAAs expression
R = 0.75, P < 0.001
SAAs expression in paratumor tissue
SAAs expression of the invasive zone
e
IHC of paratumor tissue
(Cohort 3, ICC)
SAAshigh
SAAslow
P < 0.05
Number at risk
SAAshigh 84 18 1 1
SAAslow 9 2 1 0
Months
f
RNA-seq of paratumor tissue (Cohort 5, HCC)
SAA1high
SAA1low
P < 0.001
Number at risk
SAA1high 30 26 18 11 7 0
SAA1low 129 121 102 92 54 0
Months
SAA2high
SAA2low
P < 0.001
SAA2high 45 40 26 18 13 0
SAA2low 114 107 94 85 48 0
Months
g
Protein expression
(Cohort 5, HCC)
SAAshigh
SAAslow
P < 0.01
Number at risk
SAAshigh 41 37 25 17 11 0
SAAslow 118 110 95 86 50 0
Months
h
Relative expression of Saa1 and Saa2
Saa1
Saa2
***
***
pAAV.Con
pAAV.Saas-Sh
j
CRLM model
Multiplexed IF : ARG1 SAAs F4/80 CD206 DAPI
pAAV.Con
pAAV.Saas-Sh
Multiplexed IF staining
200 μm
200 μm
50 μm
50 μm
SAAs+ARG1+ hepatocyte
F4/80+ marcophage
F4/80+ CD206+ macrophage
i
SAAs concentration (ng/ml)
***
***
Medium control
Hepatocyte supernatant (pAAV.Con)
Hepatocyte supernatant (pAAV.Saas-Sh)
k
HCC model
CRLM model
Cell subtype
F4/80+ marcophage
F4/80+ CD206+ macrophage
SAAs+ARG1+ hepatocyte
ln(number of cells)
Distant
-750~-500 μm
-500~-250 μm
-250~0 μm
0~250 μm
250~500 μm
500~750 μm
Distant
Tumor
Border
Paratumor

Supplementary information, Fig. S10. High SAAs expression in damaged hepatocytes in the invasive zone correlates with a poor prognosis in patients with liver cancer. (a-b) Overall survival (OS) curves of 10 patients with ICC from Validation Cohort 2 grouped by the *SAA1* a. or *SAA2* b. expression level in margin areas based on the bulk RNA-seq data. **c.** OS curves of 26 patients with secondary liver cancer from Validation Cohort 4 grouped based on the SAAs/ARG1 ratio determined using multiplexed IF staining. **d.** Scatter diagram showing the correlations between the SAAs expression levels in the invasive zone and paratumor tissues determined by IHC staining in 93 ICC patients from Validation Cohort 3. **e.** OS curves of 93 patients with ICC from Validation Cohort 3, grouped by their relative expression levels of SAAs in paratumor tissues as determined by IHC staining. **f.** OS curves of 159 patients with HCC from Validation Cohort 5, grouped by the relative expression levels of *SAA1* and *SAA2* in the paratumor tissues as indicated by RNA-seq data. **g.** OS curves of 159 patients with HCC from Validation Cohort 5, grouped by their relative expression levels of SAAs in paratumor tissues based on proteomic data. **h.** Relative transcriptional expression levels of SAAs in the liver tissues of mice in pAAV9-Con group or pAAV9-*Saas*-Sh group. **i.** Secreted SAAs protein levels measured by mouse SAAs enzyme-linked immunosorbent assay (ELISA) kit in the supernatant of murine primary hepatocytes cultured for 12 hours of mice in pAAV9-Con group and pAAV9-*Saas*-Sh group. **j.** Multiplexed IF staining images shows the tumor ecosystem including hepatocyte (ARG1), $SAAs^+$ hepatocyte, macrophage (F4/80) and M2 macrophage ($F4/80^+CD206^+$ cells) in invasion zone from mouse CRLM model **k.** Quantification of macrophages, M2 macrophages and $SAAs^+$ hepatocytes in different layers of the invasive zone (1000 μm in normal length) from HCC models and CRLM models. The Log-rank test was used to analyze the data in panels **a-c**, **e-g**. Student's *t*-test was used to analyze the data in panel **h** and **k**. * represents $P < 0.05$; **, represents $P < 0.01$; ***, represents $P < 0.001$.
