# Supplementary material for: An invasive zone in human liver cancer identified by Stereo-seq promotes hepatocyte–tumor cell crosstalk, local immunosuppression and tumor progression
Source: Cell Res. 2023 Jun 19;33(8):585–603. doi: 10.1038/s41422-023-00831-1 (PMC10397313; doi:10.1038/s41422-023-00831-1)
Supplement: Supplementary file 11 — Supplementary Table S1 [file 41422_2023_831_MOESM11_ESM.pdf]

Table S1. Detailed information for 53 samples used to generate 98 Stereo-seq slides and for 16 scRNA-seq samples from 23 patients with liver cancer (Discovery Cohort)

Patient ID	Sample application							
	scRNA-seq				Stereo-seq slides (sample/slide)			
	Tumor (T)	Margin area (M)	Adjacent normal (P)	Lymph Node (LN)	Tumor (T)	Margin area (M)	Adjacent normal (P)	Lymph Node (LN)
LC0						2/2	1/2	
LC1					1/2	1/2	1/2	1/2
LC2					1/2	1/1	1/2	1/2
LC3					1/2	1/2		
LC4					1/2	1/2	1/2	1/1
LC5					1/2	1/1	1/2	1/2
LC6					1/2	1/1	1/2	1/2
LC7					1/2	1/1	1/2	1/2
LC8					1/2	1/2	1/2	
LC9					1/2		1/1	
LC10						1/2	1/2	1/2
LC11	1	1	1	1		1/2		
LC12	1	1		1	1/2	1/2		1/2
LC13	1	1		1		1/2		
LC14					1/1	1/2		1/2
LC15					1/2	1/2		1/2
LC16						1/2		
LC17						1/2		
LC18							1/2	
LC19						1/2		
LC20						1/2		
LC21	1	1	1	1				
LC22	1	1						

Sample/slide means the number of sample and Stereo-seq slides from the sample or samples, respectively. The Stereo-seq slide from one sample with higher sequencing quality was selected for further analysis. There were 3 patients' samples that were analyzed by both Stereo-seq and scRNA-seq (LC11, HCC; LC12 and LC13, ICC) with gray background highlighted.
