# Supplementary material for: An invasive zone in human liver cancer identified by Stereo-seq promotes hepatocyte–tumor cell crosstalk, local immunosuppression and tumor progression
Source: Cell Res. 2023 Jun 19;33(8):585–603. doi: 10.1038/s41422-023-00831-1 (PMC10397313; doi:10.1038/s41422-023-00831-1)
Supplement: Supplementary file 12 — Supplementary Table S2 [file 41422_2023_831_MOESM12_ESM.pdf]

Table S2. The clinical and pathological information for the 23 liver cancer patients (Discovery Cohort)

Patient ID	Sex	Age	Liver cancer	Tumor size (cm)	Lymph node metastasis	Distant metastasis	Virus infection	Tumor stage
LC0	F	66	ICC	8.5×8×7.5	0	0	/	II[#]
LC1	F	65	ICC	4×4×2.5	0	1	/	IV[#]
LC2	F	50	ICC	5×5×5	0	0	/	IA[#]
LC3	M	65	ICC	3.5×2.5×2.5	0	0	HBV	IA[#]
LC4	M	37	ICC	6.5×5×4	1	0	HBV	IIIB[#]
LC5	F	67	ICC	7×3×3	0	0	/	II[#]
LC6	M	63	ICC	7×7×5	1	0	/	IIIB[#]
LC7	M	65	ICC	2.1×1.8×1.5	0	0	HBV	IA[#]
LC8	F	82	ICC	3.5×3×2	0	0	/	II[#]
LC9	M	54	ICC	6.6×4×4	1	0	HBV	IIIB[#]
LC10	M	67	ICC	7×4×4	1	0	/	IIIB[#]
LC11	M	38	HCC	10×9×9	1	0	HBV	Ib[*]
LC12	M	50	ICC	5×4×3.5	1	0	/	IIIC[#]
LC13	F	76	ICC	3.5×3.5×2.5	1	0	HBV	IIIB[#]
LC14	M	71	ICC	5.5×5×4	1	0	/	IIIB[#]
LC15	F	52	ICC	6×6×5	1	0	/	IIIB[#]
LC16	M	42	HCC	9.5×7×5	0	0	HBV	Ib[*]
LC17	F	59	HCC	4×3.3×2.8	0	0	/	Ia[*]
LC18	F	66	HCC	5×3.5×2.5	0	0	HBV	Ia[*]
LC19	M	59	HCC	6.5×5×5	0	0	/	IIIa[*]
LC20	M	59	HCC	12×11×10	0	0	HBV	IIIa[*]
LC21	M	68	ICC	4.5×4.5×4.5	1	0	/	IIIC[#]
LC22	F	69	ICC	2.5×2×1.5	0	0	/	IA[#]

#, for ICC patients, the tumor staging was according to the AJCC 8th staging system[1]; *, for HCC patients, the tumor staging was according to the CNLC staging system[2]

REFERENCES

1. Lee, A. J. & Chun, Y. S. Intrahepatic cholangiocarcinoma: the AJCC/UICC 8th edition updates. Chin Clin Oncol 7, 52 (2018).
2. Zhou, J. et al. Guidelines for the Diagnosis and Treatment of Hepatocellular Carcinoma (2019 Edition). Liver Cancer 9, 682-720 (2020).
