# Supplementary material for: An invasive zone in human liver cancer identified by Stereo-seq promotes hepatocyte–tumor cell crosstalk, local immunosuppression and tumor progression
Source: Cell Res. 2023 Jun 19;33(8):585–603. doi: 10.1038/s41422-023-00831-1 (PMC10397313; doi:10.1038/s41422-023-00831-1)
Supplement: Supplementary file 13 — Supplementary Table S3 [file 41422_2023_831_MOESM13_ESM.pdf]

Table S3. Sources of study subjects and cohorts

Biological samples	Source	Cohort
FFPE tissue blocks of primary liver cancer including: HCC (n = 53), ICC (n = 52)	Zhongshan Hospital Fudan University	Validation cohort 1 (n = 105)
Frozen samples (ICC)	Zhongshan Hospital Fudan University	Validation cohort 2 (n = 10)
FFPE tissue blocks (ICC)	Zhongshan Hospital Fudan University	Validation cohort 3 (n = 93)
FFPE tissue blocks of pan-cancer cohort including: HCC (n = 7), ICC (n = 20), and secondary liver cancer from colorectal cancer (n = 5), pancreatic cancer (n = 4), lung cancer, (n = 5), gallbladder cancer (n = 5), gastric cancer (n = 5), and ovarian cancer (n = 5)	Zhongshan Hospital Fudan University	Validation cohort 4 (n = 56)
Frozen samples (HCC)[61]	Zhongshan Hospital Fudan University	Validation cohort 5 (n = 159)
