# Supplementary material for: An invasive zone in human liver cancer identified by Stereo-seq promotes hepatocyte–tumor cell crosstalk, local immunosuppression and tumor progression
Source: Cell Res. 2023 Jun 19;33(8):585–603. doi: 10.1038/s41422-023-00831-1 (PMC10397313; doi:10.1038/s41422-023-00831-1)
Supplement: Supplementary file 14 — Supplementary Table S4 [file 41422_2023_831_MOESM14_ESM.pdf]

Table S4. Numbers of unique molecular identifiers (UMIs) and genes detected at bin50 resolution for each set of Stereo-seq data

Chip	nUMI_mean	nGenes_mean	nUMI_median	nGenes_median
LC0-M_CK1	1334	569	1178	536
LC0-M_FJ1	6964	366	598	313
LC0-M_FJ2	2730	1204	2332	1059
LC0-P_FJ3	1754	635	1634	620
LC0-P_FJ4	2369	848	2305	855
LC10-M_GB1	1102	613	806	516
LC10-M_GB2	1272	757	995	675
LC10-LN_GB3	3075	1193	2054	973.5
LC10-LN_GB4	1912	897	1086	669
LC10-P_GC3	2543	871	2231	822
LC10-P_GC4	2253	981	2130	967
LC11-M_GF1	2490	1028	2038	952
LC11-M_GF2	3231	1367	3052	1367
LC12-M_GF3	4206	1655	3659	1573
LC12-M_GF4	4385	1897	4121	1866
LC12-LN_GJ1	1448	686	1145	642
LC12-LN_GJ2	950	524	772	468
LC12-T_GJ5	1484	691	893	530
LC12-T_GJ6	1479	695	1085	588
LC13-M_GF5	4642	1669	4165	1562
LC13-M_GF6	4426	1810	4349	1677
LC14-M_GF7	1412	703	1093	624
LC14-M_GF8	2106	1046	1924	1032
LC14-LN_GJ3	589	380	489	332
LC14-LN_GJ4	959	591	805	530
LC14-T_GJ8	859	415	538	312
LC15-M_GK1	1764	694	1434	613
LC15-M_GK2	2706	995	2360	884
LC15-LN_GK3	1452	685	1146	609
LC15-LN_GK4	1774	912	1537	858
LC15-T_GK5	3136	1062	3012	1085
LC15-T_GK6	2863	1089	2695	1092
LC1-M_FG5	963	467	804	439
LC1-M_FG6	923	438	766	389
LC1-LN_FA1	1174	659	1035	647
LC1-LN_FA2	1438	806	1361	795
LC1-P_FK1	1655	539	1661	560
LC1-P_FK2	2860	888	2861	929
LC1-T_FG7	714	392	517	319
LC1-T_FG8	1077	477	711	379
LC2-M_DU1	1261	638	1132	611

LC2-LN_FK3	2287	1150	2363	1202
LC2-LN_FK4	2444	1108	2443	1132
LC2-P_FL3	1816	693	1820	711
LC2-P_FL4	1899	712	1834	718
LC2-T_FL1	1697	847	1335	751
LC2-T_FL2	1604	755	1188	645
LC3-M_FW5	1992	1048	1819	1010
LC3-M_FW6	976	597	855	551
LC3-T_FW7	1249	765	954	654
LC3-T_FW8	1393	787	1007	651
LC4-M_FE7	2347	975	2141	972
LC4-M_FE8	718	389	556	329
LC4-LN_FC1	1354	709	1179	670
LC4-P_FG3	1202	486	1134	476
LC4-P_FG4	1986	709	1899	707
LC4-T_FG1	1487	613	863	444
LC4-T_FG2	2401	989	1364	742
LC5-M_DU3	1591	796	1534	814
LC5-LN_FA3	2250	1008	2179	995
LC5-LN_FA4	2471	1072	2564	1092
LC5-P_FE1	1529	643	1453	632
LC5-P_FE2	1186	542	993	503
LC5-T_FD3	995	547	728	462
LC5-T_FD4	953	531	654	428
LC6-M_DW1	1173	594	983	576
LC6-LN_FD1	623	429	498	354
LC6-LN_FD2	854	517	779	484
LC6-P_FE3	1739	685	1644	667
LC6-P_FE4	3216	1053	3166	1067
LC6-T_FE5	1085	612	755	489
LC6-T_FE6	831	510	575	402
LC7-M_DW3	1404	667	1187	628
LC7-LN_FM1	1165	626	967	599
LC7-LN_FM2	1295	632	1149	624
LC7-P_FM5	2605	846	2382	812
LC7-P_FM6	1127	449	990	429
LC7-T_FM3	1339	648	875	512
LC7-T_FM4	1450	634	955	512
LC8-M_FT1	1994	926	1564	768
LC8-M_FT2	3608	1440	3034	1267
LC8-P_FV3	1457	479	1432	462
LC8-P_FV4	783	397	735	377
LC8-T_FV1	2256	827	1812	718
LC8-T_FV2	1740	824	1427.5	724

LC9-P_FV8	1183	445	1110	433
LC9-T_FV5	694	511	601	470
LC9-T_FV6	653	468	568	430
LC16-M_HA1	3103	1191	2812.5	1118
LC16-M_HA2	2852	1178	2619	1118
LC17-M_HA3	2140	920	1979	875
LC17-M_HA4	1778	887	1702	871
LC18-P_HA5	1937	772	1903	780
LC18-P_HA6	1430	650	1399	656
LC19-M_GM1	1430	661	1159	585
LC19-M_GM2	2109	886	1789	808
LC20-M_GM3	2667	1126	2581	1105
LC20-M_GM4	2234	921	2145	894
