# Supplementary material for: An invasive zone in human liver cancer identified by Stereo-seq promotes hepatocyte–tumor cell crosstalk, local immunosuppression and tumor progression
Source: Cell Res. 2023 Jun 19;33(8):585–603. doi: 10.1038/s41422-023-00831-1 (PMC10397313; doi:10.1038/s41422-023-00831-1)
Supplement: Supplementary file 15 — Supplementary Table S5 [file 41422_2023_831_MOESM15_ESM.pdf]

Table S5. Selected marker genes for different cell subtypes

Cell types	Cell subtypes	Gene list
T cells	resident T cells	*RUNX3, NR4A1, CD69, CXCR6, NR4A3*
	cytotoxic T cells	*PRF1, IFNG, GNLY, NKG7, GZMB, GZMA, CST7, TNFSF10*
	exhausted T cells	*CTLA4, HAVCR2, LAG3, PDCD1, TIGIT*
	costimulatory T cells	*ICOS, CD226, TNFRSF14, TNFRSF25, TNFRSF9, CD28*
	naïve T cells	*CCR7, TCF7, LEF1, SELL*
B cells	naïve B cells	*MS4A1, CD19, CD22, TCL1A, CD83, BANK1, CD79A*
	plasma B cells	*MZB1, IGLL1, IGLL5, SSR4, JCHAIN, IRF4, SDC1, XBP1, PRDM1*
	memory B cells	*FCRL4, CCR1, CD27, CD44, GPR183, CD69, CXCR4, CCR7, KLF2*
	germinal center B cells	*AICDA, RGS13, GCSAM, BCL6, NANS, CD81, CD38*
	HLA-II	*HLA-DRB1, HLA-DQB1, HLA-DPB1, HLA-DRA, HLA-DQA1, HLA-DPA1, HLA-DRB5, HLA-DRB4, HLA-DQA2, HLA-DRB3, HLA-DOA, HLA-DMA, HLA-DMB, HLA-DQB2, HLA-DOB*
macrophages	anti-inflammatory	*IL1RN, IL10, IL4, IL11, IL13, TGFB1, TNFRSF1A, TNFRSF1B, IL1R2, IL18BP, CCL17, CCL18, CCL22, CCL24*
	pro-inflammatory	*IL1B, TNF, CCL2, CCL3, CCL5, CCL7, CCL8, CCL13, IL6, IL12, IL23, CXCL9, CXCL10*
